# Identification of sRNAs expressed by the human pathogen *Neisseria gonorrhoeae* under disparate growth conditions

**DOI:** 10.3389/fmicb.2014.00456

**Published:** 2014-08-28

**Authors:** Ryan McClure, Brian Tjaden, Caroline Genco

**Affiliations:** ^1^Department of Medicine Section of Infectious Disease, Boston University School of MedicineBoston, MA, USA; ^2^Department of Microbiology, Boston University School of MedicineBoston, MA, USA; ^3^Department of Computer Science, Wellesley CollegeWellesley, MA, USA

**Keywords:** small RNA, *Neisseria gonorrhoeae*, RNA-seq, host cell, iron

## Abstract

In the last several years, bacterial gene regulation via small RNAs (sRNAs) has been recognized as an important mechanism controlling expression of essential proteins that are critical to bacterial growth and metabolism. Technologies such as RNA-seq are rapidly expanding the field of sRNAs and are enabling a global view of the “sRNAome” of several bacterial species. While numerous sRNAs have been identified in a variety of both Gram-negative and Gram-positive bacteria, only a very small number have been fully characterized in the human pathogen *Neisseria gonorrhoeae*, the etiological agent of the STD gonorrhea. Here we present the first analysis of *N. gonorrhoeae* specifically focused on the identification of sRNAs through RNA-seq analysis of the organism cultured under different *in vitro* growth conditions. Using a new computational program, Rockhopper, to analyze prokaryotic RNA-seq data obtained from *N. gonorrhoeae* we identified several putative sRNAs and confirmed their expression and size through Northern blot analysis. In addition, RNA was collected from four different growth conditions (iron replete and deplete, as well as with and without co-culture with human endocervical cells). Many of the putative sRNAs identified shoed varying expression levels relative to the different growth conditions examine or were detected only under certain conditions but not others. Comparisons of identified sRNAs with the regulatory pattern of putative mRNA targets revealed possible functional roles for these sRNAs. These studies are the first to carry out a global analysis of *N. gonorrhoeae* specifically focused on sRNAs and show that RNA-mediated regulation may be an important mechanism of gene control in this human pathogen.

## Introduction

*Neisseria gonorrhoeae*, the causative agent of the STD gonorrhea, is one of the most proliferative bacterial agents in the United States and abroad and is beginning to show an alarming resistance to conventional antibiotics (Camara et al., 2012; Allen et al., 2013). During acute infection, this pathogen, like many other bacterial organisms, must quickly adapt to changing environmental conditions that include a host-mediated inflammatory response and the presence of other organisms (Nikolaitchouk et al., 2008) as well as variations in oxygen and iron levels (Newkirk, 1996; Agarwal et al., 2008; Ma et al., 2012). Such rapid adaptations require sophisticated mechanisms of bacterial gene regulation. In the gonococcus, regulation of gene expression can occur via alternative sigma factors (Laskos et al., 2004; Gunesekere et al., 2006), frameshift and promoter mutations (Stern et al., 1986; Banerjee et al., 1998; Henderson et al., 1999) as well as more classical DNA binding proteins. However, one mechanism of regulation that has been described in other organisms but which is only beginning to be understood in *N. gonorrhoeae* is mediated by regulatory small RNA (sRNA) transcripts.

Bacterial sRNA molecules are analogous to eukaryotic microRNAs and act as post-transcriptional regulators, affecting the translation and stability of mRNA targets or regulating proteins directly (Repoila and Darfeuille, 2009; Waters and Storz, 2009). Most sRNAs that function to regulate mRNAs operate by binding to their targets in the 5′ untranslated region (UTR) through short regions of complementarity to affect their translation or stability. In many cases, sRNA binding leads to a decrease in translation of target genes (Vanderpool and Gottesman, 2004; Udekwu et al., 2005; Heidrich et al., 2007), yet under certain conditions, sRNAs can cause strand shifting in target mRNAs to open up ribosome binding sites, leading to increased expression (Soper et al., 2010). Nearly all trans-acting sRNAs are expressed from intergenic (IG) regions or are expressed as antisense transcripts opposite a known protein-coding gene. A majority of sRNAs end transcription using a *rho*-independent terminator (RIT), an inverted repeat which forms an RNA hairpin loop followed by a U-rich sequence that stalls transcription. These characteristics have been used extensively to perform global searches for sRNAs through *in silico* analysis of bacterial genomes (Chen et al., 2002; Panek et al., 2008; Perez et al., 2009).

sRNAs generally act as post-transcriptional regulators and as such are regulated themselves via a variety of stimuli. During bacterial growth, proper homeostasis of intracellular iron levels is mediated, in part, by regulatory sRNAs. Perhaps the most well studied example is the *E. coli* sRNA RyhB. This sRNA is negatively regulated by iron and when expressed leads to repression of the transcripts for *sodB* and *sdhC/A* (Masse and Gottesman, 2002). Homologs of RyhB have been discovered in a large number of pathogenic bacteria including *Shigella* species, *Vibrio cholera*, *Salmonella enterica*, *Y. pestis*, and *N. gonorrhoeae* (Davis et al., 2005; Mey et al., 2005; Oglesby et al., 2005; Murphy and Payne, 2007; Padalon-Brauch et al., 2008; Ducey et al., 2009; Deng et al., 2012). A large number of sRNAs have also been identified that have roles in pathogenic mechanisms of bacteria. In *Pseudomonas aeruginosa* the action of a translational repressor protein RsmA is regulated by two sRNAs, RsmY, and RsmZ. These sRNAs act as decoy targets for the DNA binding sequence of RsmA (Valverde et al., 2004) acting to sequester the protein allow expression of virulence genes (Pessi et al., 2001; Kay et al., 2006; Mulcahy et al., 2008). In *V. cholera*, quorum sensing is a crucial element of pathogenesis and is regulated by four redundant sRNAs termed quorum regulated RNA (Qrr)1-4 (Bardill and Hammer, 2012). These sRNAs require the sRNA cofactor Hfq and strains lacking Qrr sRNAs are severely impaired in mouse models of infection (Miller et al., 2002; Zhu and Mekalanos, 2003; Bardill et al., 2011).

While sRNAs have been well known for decades and a multitude of examples have been described in *E. coli* and other organisms, regulatory mechanisms using sRNAs in Neisseria species are only beginning to be analyzed. Our laboratory identified the first sRNA in Neisseria, Neisserial RNA responsive to Fe (NrrF), which is regulated by iron availability via the Fur protein (Mellin et al., 2007). Once transcribed, NrrF goes on to negatively regulate translation of the *sdhC/A* mRNA transcripts. In depth analysis of a second sRNA in *N. gonorrhoeae* that acts to increase antigenic variability of pilin structures of the gonococcus has been carried out by Cahoon and Seifert (2013). Other sRNAs have also been found but their specific targets and phenotypic effects remain to be elucidated (Isabella and Clark, 2011). In several other bacterial species RNA sequencing has been used to identify new sRNAs (Gomez-Lozano et al., 2012; Kroger et al., 2012; Lee et al., 2013; Soutourina et al., 2013). These approaches generally involve the sequencing of total or size selected RNA to identify sRNAs. Such studies have been very successful and have identified hundreds of putative transcripts that may function as post-transcriptional regulators is a diverse array of bacteria. While a small number of sRNAs have been discovered in *N. gonorrhoeae* there has not yet been a similar global overview of novel sRNAs in *N. gonorrhoeae* to date. In the current study we present the results of an RNA-seq analysis carried out on size selected RNA samples from *N. gonorrhoeae* grown *in vitro*. We identify and confirm a number of novel sRNAs in *N. gonorrhoeae* under these conditions. In addition, subsequent experiments delineate how these sRNAs are regulated, how the profile of expressed sRNAs changes with variable growth conditions and posits possible targets of these sRNAs. These experiments represent the first global search for sRNAs of the gonococcus and suggest that sRNAs, as in other organisms, may play a large role in genetic regulation in *N. gonorrhoeae*.

## Materials and methods

### Bacterial strains and culture conditions

*N. gonorrhoeae* F62 was the strain used in this study (Schneider et al., 1982). We chose to use this strain as it has been used in our laboratory successfully in the past and is particularly amenable to transformation. *N. gonorrhoeae* was plated onto GCB agar plates and grown for 16–18 h at 37°C in 5% CO_2_. To identify novel sRNAs, *N. gonorrhoeae* was resuspended in Chemically Defined Media (CDM) (Grifantini et al., 2003) at an O.D._600_ of 0.1 and grown until mid-log phase (O.D._600_ of ~0.25) before being split into two cultures. To constitute an iron replete condition, ferric nitrate was then added to one culture at a final concentration of 100 μM. To constitute an iron deplete condition, desferal, an iron chelator, was added to a second culture at a final concentration of 100 μM. RNA was isolated 90 min later using TRIzol according to the manufacturer's instructions. Due to prohibitive costs at the beginning of these studies RNA from high and low iron conditions was combined without barcoding before being sequenced to identify sRNAs. Isolation of RNA for experiments aimed at determining how iron leads to changes in sRNA expression were carried out as above except that high and low iron conditions were maintained for 60 min prior to RNA isolation. In addition, RNA from high and low iron conditions was barcoded prior to sequencing so that iron-mediated regulation could be observed.

### *In vitro* incubation of human cells with *N. gonorrhoeae*

Epithelial cells End/E6E7 (Fichorova et al., 1997) were seeded in six-well tissue culture plates (5 × 10^5^ cells/well) and allowed to grow to confluence (10^6^ cells/well) in antibiotic-free KSFM, a process taking 24–48 h. Concurrently, *N. gonorrhoeae* F62 was plated on GCB plates and grown as above for 17 h. Bacterial cells were removed from plates and resuspended in KSFM. Media was then removed from wells containing End/E6E7 and KSFM containing *N. gonorrhoeae* was then added to each well at an MOI of 100. An equal amount of *N. gonorrhoeae* was also incubated with KSFM alone to constitute a negative control. A sample of the original *N. gonorrhoeae* inoculum was also plated and CFU counted to determine exact MOI. Plates were then incubated at 37° C in 5% CO_2_ and 2 h later media was removed from End/E6E7 incubated with *N. gonorrhoeae* and wells were washed 3× with 2.5 mL of PBS to remove all *N. gonorrhoeae* cells which were not adherent to or within host cells. PBS was removed and TRIzol was then added to each well and plates were agitated gently for 10 min. TRIzol from each well was then transferred to a 2 mL tube and RNA was isolated according to the manufacturer's instructions. For *N. gonorrhoeae* incubated alone in media the contents of each well were transferred to a 2 mL tube and were centrifuged for 3 min at 3000 rpm. The resulting bacterial pellet was resuspended in 1 mL of TRIzol and RNA was extracted.

### *Neisseria gonorrhoeae* RNA-seq experiments

Representative samples of RNA were isolated from *N. gonorrhoeae* as described above and DNase treated using TURBO DNase (Ambion) according to the manufacturer's instructions to remove contaminating DNA. Before subsequent sequencing all RNA was visualized on a 1.5% agarose gel to confirm RNA quality and integrity. Identification of sRNAs and analysis of regulation of sRNAs by iron or incubation with epithelial cells was carried out as follows. To identify sRNAs, RNA grown under iron replete or deplete conditions for 90 min was first gel purified to enrich for RNA transcripts 50–250 nucleotides in length. RNA was electrophoresed for 1 h at 200 V through a 15% TBE-Urea gel (Invitrogen) in TBE buffer before being extracted. A cDNA library was then generated from the enriched RNA using Illumina's sRNA Sample Preparation Kit and samples were sequenced on an Illumina GAIIx without barcoding. To analyze the role of iron and epithelial cells on sRNA expression a 3 μg sample of *N. gonorrhoeae* RNA incubated under iron replete/deplete conditions for 60 min. or with/without epithelial cells for 2 h. was depleted of ribosomal RNA using the MicrobeEXPRESS kit (Ambion) according to manufacturer's instructions. A cDNA library of the resulting mRNA was then prepared using BioChain's Directional mRNA Sample Prep kit according to the manufacturer's instructions. In the experiments described here sRNAs were first identified (via analysis of size-selected RNA without barcoding) and identified sRNAs were then analyzed to determine regulation through additional RNA-seq experiments (under various conditions and with barcoding). Iron regulation and epithelial cell incubation experiments were part of studies examining the response of the entire transcriptome of *N. gonorrhoeae* to these stimuli and thus RNA was not size selected before sequencing. All libraries analyzed on an Agilent Bioanalyzer before sequencing to confirm library quality and were run using 36–72 basepair reads on an Illumina GAIIX machine. Two biological replicates were performed examining sRNAs specifically or examining iron regulation of sRNAs. One biological replicate was carried out examining sRNAs regulated via incubation with E6/E7 cells.

### Analysis of raw RNA sequencing data

Data resulting from the above RNA-seq experiments was analyzed with the tool Rockhopper (McClure et al., 2013) and a summary of the analysis methods is provided below. Reads were aligned to the *N. gonorrhoeae* FA 1090 genome. While the F62 genome has been sequenced, it has not yet been fully assembled. Therefore, the FA1090 genome was chosen as a template to align reads to. We have used the FA1090 genome for design of primers and probes that were then used with F62 RNA and DNA and have encountered no problems. Several studies have shown the high degree of similarity between the F62 and FA1090 genomes (Snyder et al., 2004; Jordan et al., 2005) indicating that alignment of F62 reads to the FA1090 genome poses no major impediment to downstream analysis. Using an approach similar to that of Bowtie2 (Langmead and Salzberg, 2012) a Burrows-Wheeler index based on the full-text minute space was created for the FA1090 genome. Using the index, an alignment to the genome was attempted for each read. If a read did not align exactly, then seed regions of the read were aligned to the genome. These seed alignments were extended with a dynamic program that employed a quality aware scoring function based on the error probability of each sequencing read nucleotide to ensure the highest quality alignment of the read to the genome. Seed regions were constrained to be no less than one third the length of the read and inexact alignments of a read to the genome were allowed to contain up to a threshold number of mismatches, insertions, and deletions, where the threshold was set to 15% of the length of the read. Following alignment of the sequencing reads to the genome, reads from each experiment were normalized by upper quartile normalization (Bullard et al., 2010) and transcripts were assembled, first, by identifying a set of transcript seeds consisting of annotated genes and high confidence novel transcripts and, then, by extending the seeds using a Bayesian approach to identify more precise transcript boundaries. Transcript abundance levels were quantified using the Reads Per Kilobase per Million mapped reads (RPKM) measure except that instead of normalization by total read counts in each experiment, upper quartile normalization was used to increase robustness of the transcript abundance estimates. This approach eliminated potential skewing of the normalization process due to large numbers of gonococcal genes that may have very low or no expression. Other studies that have used upper quartile normalization have found that it has some of the best concordance with qRT-PCR data (Bullard et al., 2010).

### Northern blot analysis of sRNAs

For Northern blot analysis, 20–30 μg of bacterial RNA along with a ssRNA ladder were first denatured with glyoxal dye at 50°C for 30 min. Denatured RNA was electrophoresed through a native 1.5% agarose gel at 90 V for 60 min. Following confirmation that RNA was intact via viewing with UV light, RNA was transferred to positively charged nitrocellulose membranes for 2.5–3 h using passive transfer with 20X SSC. After transfer, RNA was cross linked to membranes with UV light and pre-incubated with OligoHyb Buffer (Ambion) for 45–60 min at 37°C with rotation. Simultaneously, ~50 nucleotide oligo probes were labeled with [α-^32^P] ATP using T4 PNK for 60 min at 37°C followed by 2 min at 90°C to inactivate the PNK (Table S1). Membranes were then pre-incubated for 30 min in OligoHyb before each probe was diluted with a further 1 mL of OligoHyb and placed in tubes containing each membrane. Membranes were incubated with probes at 37°C with rotation overnight. Following probe hybridization, membranes were washed twice at RT with 2X SSC containing 0.1% SDS. Membranes were exposed to x-ray film overnight at −80°C and developed. Size of sRNAs was determined by plotting the base 10 logarithm of the size of each ladder marker against distance traveled in the gel on semi-log paper. sRNAs were then sized using the resulting standard curve.

### Primer extension analysis

For primer extension analysis, 10 μg of bacterial RNA was incubated with an [α-^32^P] ATP radiolabeled oligonucleotide probe at varying temperatures corresponding to the probe's melting temperature. Probes (Table S1) were labeled using T4 PNK for 60 min. at 37°C followed by 2 min at 90°C to inactivate the PNK. Following probe hybridization to bacterial RNA the probes were extended using reverse transcriptase for 1 h at 41°C. Single stranded DNA (ssDNA) products were then electrophoresed through an 8% TBE-Urea gel along with a radiolabeled ssDNA ladder. Gels were exposed to x-ray film overnight at −80°C and developed. Size of primer extension products was determined by plotting the base 10 logarithm of each ladder marker against distance traveled in the gel on semi-log paper. Primer extension products were sized using the resulting standard curve.

## Results

### Identification and confirmation of sRNAs in *N. gonorrhoeae*

All samples were sequenced on an Illumina GAIIx machine and aligned to the FA1090 genome with Rockhopper, a new program designed to analyze prokaryotic RNA-seq data (McClure et al., 2013). Alignment results from each experimental condition are shown in Table 1. Size selected RNA showed by far the most amount of RNA aligning to non-annotated portions of the genome. This is likely a result of gel electrophoresis filtering out mRNAs and larger rRNAs and allowing for a greater proportion of sRNAs in the sequenced sample. The remaining alignment to rRNA in these samples likely reflects presence of the 5S transcript. The gonococcal 5S transcript is 119 nucleotides in length and thus would likely be selected along with sRNAs during gel electrophoresis. There was little difference in the alignment of the iron replete and deplete samples. However, RNA isolated from *N. gonorrhoeae* during incubation with endocervical cells or in KSFM media alone showed higher amounts of RNA aligning to non-annotated portions of the genome. This may reflect the large number of sRNAs expressed under these conditions compared to iron related conditions, particularly during incubation of *N. gonorrhoeae* with endocervical cells (see below). These results indicate that a size selection step prior to sequencing may increase the amount of sRNAs detected through depletion of much of the coding RNA expressed in the cell.

**Table 1:** **Summary of cDNA alignment**.

**Condition**	**Aligned reads**	**Percentage of reads aligning to rRNA**	**Percentage of reads aligning outside ORFs**
Size selected (Rep. 1)	19068325	0.64	0.15
Size selected (Rep. 2)	30681113	0.28	0.23
Whole transcriptome-iron replete (Rep. 1)	7634630	0.84	0.03
Whole transcriptome-iron replete (Rep. 2)	2617049	0.85	0.03
Whole transcriptome-iron deplete (Rep. 1)	2168361	0.65	0.05
Whole transcriptome-iron deplete (Rep. 2)	4983187	0.84	0.02
Whole transcriptome-w/endocervical cells	1223115	0.65	0.08
Whole transcriptome-w/media alone	17979679	0.51	0.07

Following RNA sequencing of size selected samples obtained from *N. gonorrhoeae* grown *in vitro*, subsequent analysis focused on the identification of possible transcripts that corresponded to sRNAs. Our first analysis focused on two separate conditions: growth under iron-replete conditions (100 μM ferric nitrate) or under iron-deplete conditions (100 μM desferal, an iron chelator). As described in the Methods size-selected RNA isolated from these conditions was initially sequenced together without barcoding. Transcripts showing expression were identified within IG regions or antisense to protein coding genes. These parameters generated a list of 280 putative sRNA transcripts that were expressed under either iron replete or deplete conditions (Table S2). Since sRNAs are generally between 50 and 250 nucleotides in length any RNA transcripts that were 30 nucleotides or smaller in length were removed from further analysis. This left a list of 232 sRNA candidates.

**Figure 1:**
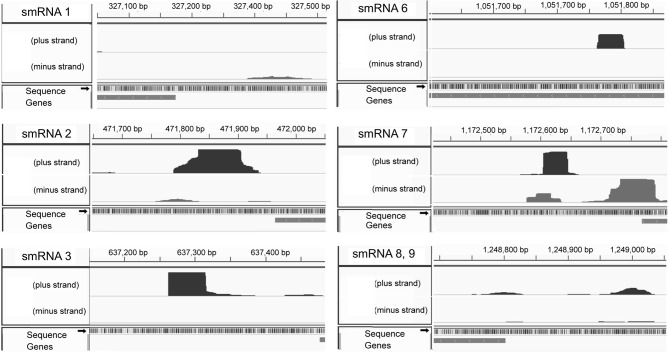
**Expression as determined by Rockhopper analysis.** Strand specificity are shown on the left side of each and genomic location on the top. The shape of each pixelated column indicates expression level and coordinates of each sRNA. smRNA 8 and 9 are located in close genetic proximity and are shown in one image.

**Figure 2:**
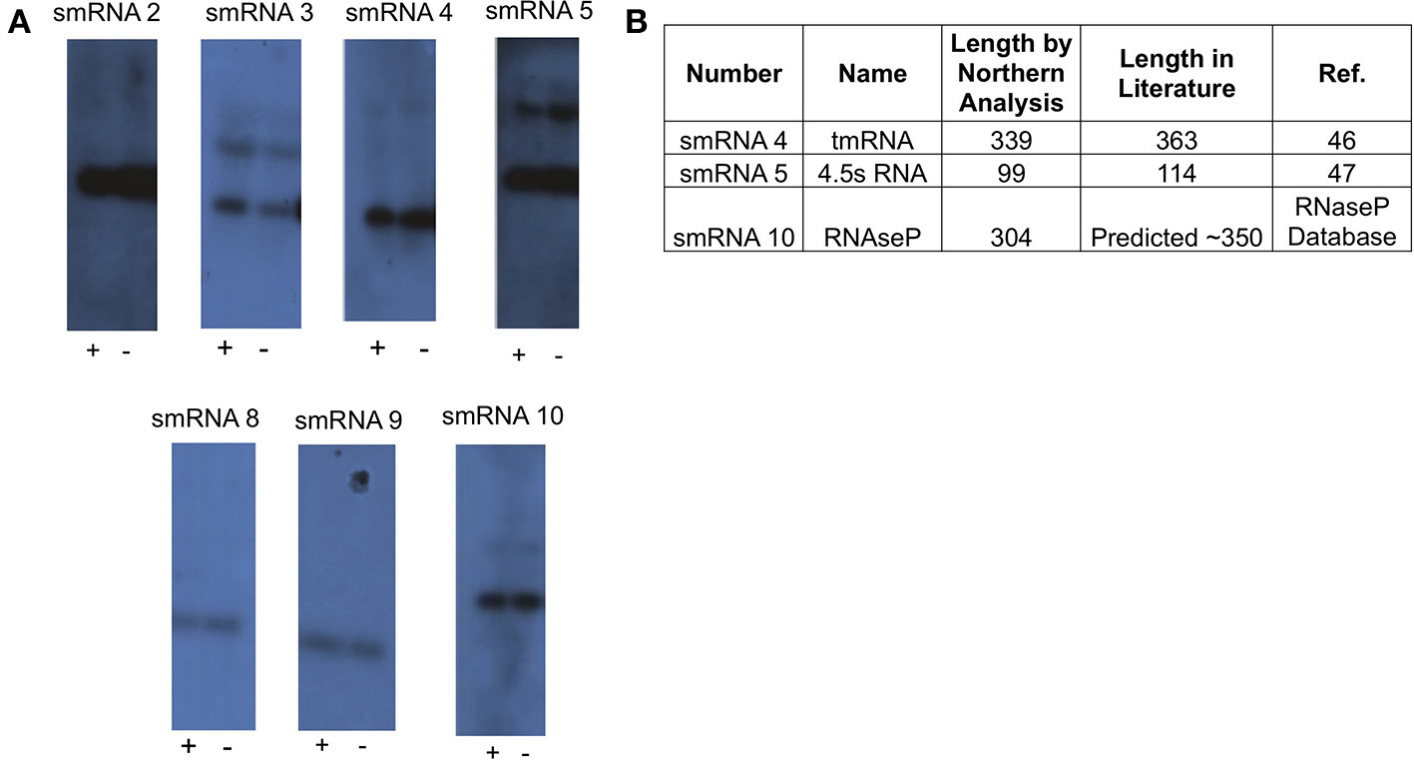
**Northern blot analysis of novel sRNAs. (A)** A 20 μg sample of RNA was analyzed via Northern Blot analysis to confirm expression and size of putative sRNAs. All putative sRNAs that were examined were confirmed. sRNA expression was also tested under conditions related to those used for RNA-seq. Growth for 90 min under iron replete conditions (indicated by “+”), and growth for 90 min under iron deplete conditions (indicated by “−”). Experiments were performed three times and a representative film is shown. **(B)** Table showing previously known non-coding sRNAs that were also found in this study.

From this list of sRNAs, a subset of 10 (Table 2) were chosen for expression confirmation via Northern blot analysis, the expression levels of these sRNAs was visualized using the Integrated Genome Viewer (IGV) and Rockhopper (Figure 1). Of the 10 sRNAs chosen, seven were detected by Northern blot analysis (Figure 2A). All of the seven sRNAs analyzed by Northern blot analysis were less than 500 nucleotides in size, suggesting that they are not likely to be UTRs of flanking protein coding genes (Figure 2A and Table 1). In addition, a comparison was performed between the sizes of sRNAs as predicted by Northern blot analysis vs. by RNA-seq analysis. The average ratio of sRNA sizes predicted by RNA-sequencing compared to sRNA sizes confirmed by Northern blot analysis was 0.57. Generally, sRNA sizes predicted by RNA-seq analysis were smaller than sRNA sizes as determined by Northern blot analysis.

**Table 2:** **Small RNAs examined via Northern blot analysis**.

Name^*^	**Transcription start^#^**	**Transcription stop^#^**	**Transcript size determined by RNA-seq**	**Transcript size determined by Northern blot analysis**	**Contains a RIT^∥^**	**Expression ^&^**
smRNA1	324576	324615	44	N/A	Yes	919
smRNA2	471816	471905	90	89	No	21216
smRNA3	637363	637414	52	160	Yes	16150
smRNA4	863131	863310	180	339	No	24422
smRNA5	870849	870808	42	99	No	3707
smRNA6	1051765	1051802	38	N/A	No	709
smRNA7	1172605	1172644	40	N/A	Yes	608
smRNA8	1248708	1248738	31	124	Yes	454
smRNA9	1248894	1248928	35	72	Yes	499
smRNA10	1924430	1924767	338	304	Yes	13282

* Name of the putative sRNA is shown.

# Transcription start and stop sites are shown according to the Neisseria gonorrhoeae FA1090.

∥ Whether the putative sRNA contains a Rho-Independent Terminator as determined by TransTerm is shown.

& Expression values in RPKM are shown.

In order to determine if any of the sRNAs identified were likely to be orthologs of sRNAs identified in other organisms, we queried the Rfam database (Burge et al., 2013) and found that three (smRNA 4, 5, and 10) were likely to be members of sRNA families that have been previously characterized in *Neisseria*. smRNA 4, a tmRNA, is a short RNA transcript that has been implicated in the processing of truncated polypeptides. tmRNA acts to remove polypeptides which have been synthesized as a result of stalled translation machinery (Keiler et al., 1996; Huang et al., 2000). smRNA 5 corresponds to 4.5S RNA, part of an RNA-protein complex that targets proteins for transport to the cytoplasmic membrane. Previous investigation of 4.5S RNA in *N. gonorrhoeae* suggested a size of 114 nucleotides (Frasz and Arvidson, 2003). This is in proximity to our experimentally determined size of 99 nucleotides by Northern blot analysis (Table 2). Finally, our analysis detected the RNA portion of RnaseP, MI RNA (designated as smRNA 10). This transcript has not been studied previously in *N. gonorrhoeae* but it has a predicted size of ~350 nucleotides in the RnaseP Database (Brown, 1999). This is in accordance with our Northern blot and RNA-seq analysis that demonstrated this RNA transcript to be 304 and 338 nucleotides in length, respectively. The identification of several known sRNAs thus validated the results of obtained by RNA-seq and Northern blot analyses (Figure 2B). Beyond the 10 sRNAs evaluated by Northern blot analysis, we determined if other putative sRNAs identified by our RNA-seq experiments corresponded to previously characterized sRNAs. Both NrrF and the TPP riboswitch were identified by our RNA-seq analysis (Serganov et al., 2006; Mellin et al., 2007; Ducey et al., 2009). However, we did not detect expression of a previously identified sRNA shown to be involved in pilin antigenic variation in Neisseria species. It is possible that this sRNA was expressed at very low levels as shown by previous RNA-seq experiments in *N. meningitidis* and the lack of detection of this sRNA by Northern blot analysis by other groups (Cahoon and Seifert, 2013). It is also possible that certain environmental stimuli are required for expression of this sRNA that were not present in these experiments (Cahoon and Seifert, 2013).

In addition to this overlap with other organisms the other sRNAs in our original list of 10, even those not confirmed by Northern blot, (smRNA 1, 2, 3, 4, 7, 8, 9) were examined using BLAST to determine if they were unique to *N. gonorrhoeae*. smRNA 1 was 100% identical in all *N. gonorrhoeae* sequenced strains and a homolog showing 80–90% identity was found in strains of *N. meningitidis*. smRNA 2 in contrast was unique to *N. gonorrhoeae* FA1090, no similar sequences could be found in other organisms including other strains of *N. gonorrhoeae*. smRNA 3, and smRNA 6 were found in all sequenced strains of *N. gonorrhoeae* but homologs were not detected in *N. meningitidis* or other bacteria. smRNA 7, 8, and 9 were more widespread and could be found in all strains of *N. gonorrhoeae*, *N. meninigitidis* as well as *Neisseria lactamica*.

### Genomic location and predicted structure of sRNAs

**Figure 3:**
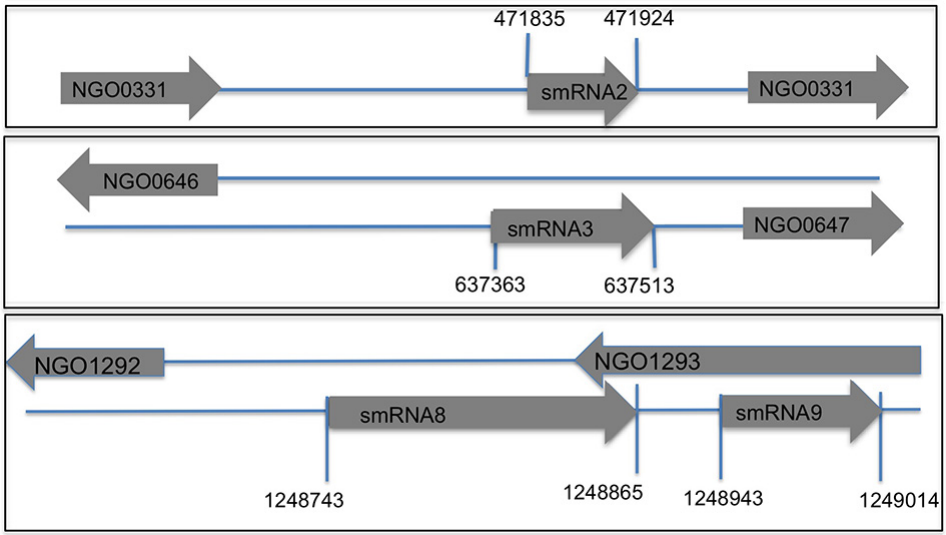
**Genomic location of sRNAs in *N. gonorrhoeae* genome FA1090.** Using transcriptional start sites and Northern blot analysis data (total size of a sRNA) the putative genomic coordinates of transcription were determined. Each novel sRNA that was confirmed by Northern blot analysis is shown with start and stop sites of transcription. Directionality of the sRNA and the flanking genes is indicated by the arrow (pointing right is transcription from the positive strand and left is transcription from the negative strand). A subset of sRNAs are antisense to known protein coding genes.

**Figure 4:**
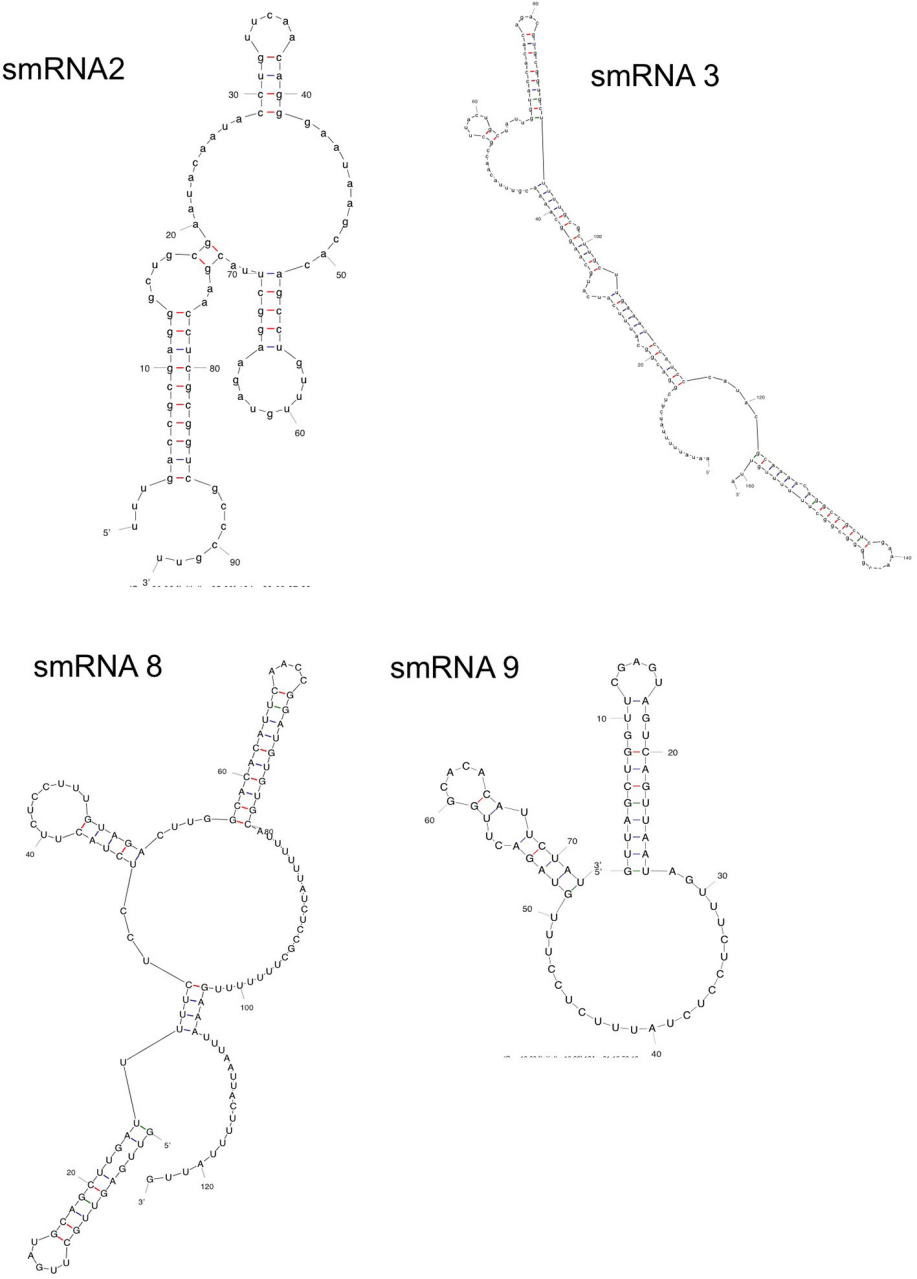
**Secondary structure of sRNAs.** mFold analysis was carried out on novel sRNAs to determine their lowest free energy secondary structures.

Following confirmation of the size and expression of each sRNA we used the transcriptional start sites predicted by Rockhopper in conjunction with Northern blot analysis to determine the genomic locations of the four novel sRNAs that had not been previously characterized. The determination of both sRNA transcriptional start site and size allowed putative genomic coordinates of each sRNA (Figure 3), thus providing a likely sRNA sequence. We also performed primer extension on a subset of the identified sRNAs. For two of the sRNAs (smRNA 8 and 9), primer extension analysis revealed that transcriptional start sites as determined by RNA-seq corresponded exactly with that determined by primer extension (Figure S1). This strong correlation between RNA-seq and primer extension increased our confidence in the transcriptional start sites of other sRNAs determined by RNA-seq analysis. Other experiments performed by our group have also confirmed the ability of Rockhopper to predict transcriptional start sites from RNA-seq data (McClure et al., 2013). Since most sRNAs act as post-transcriptional regulators of mRNAs through base pairing, characterization of the genomic location of a sRNA and, thus, its sequence, will aid in defining the mRNA targets of its regulation. For each sRNA whose sequence we were able to precisely determine, we used mFold (Zuker, 2003) to determine the lowest free energy secondary structure of the sRNA (Figure 4). While the lowest free energy structure may not correspond to the structural conformation adopted by the sRNA under all conditions, it can serve as a general starting point for structural analysis and target identification. Several of the sRNAs in question contained large single stranded regions that were rich in adenosine and uridine. In other bacteria, such unstructured AU rich regions have been shown to be putative binding sites for the common sRNA protein cofactor Hfq (Link et al., 2009). The presence of these sites suggests that these sRNAs may bind to Hfq as a required cofactor.

### Regulatory patterns of sRNAs

**Figure 5:**
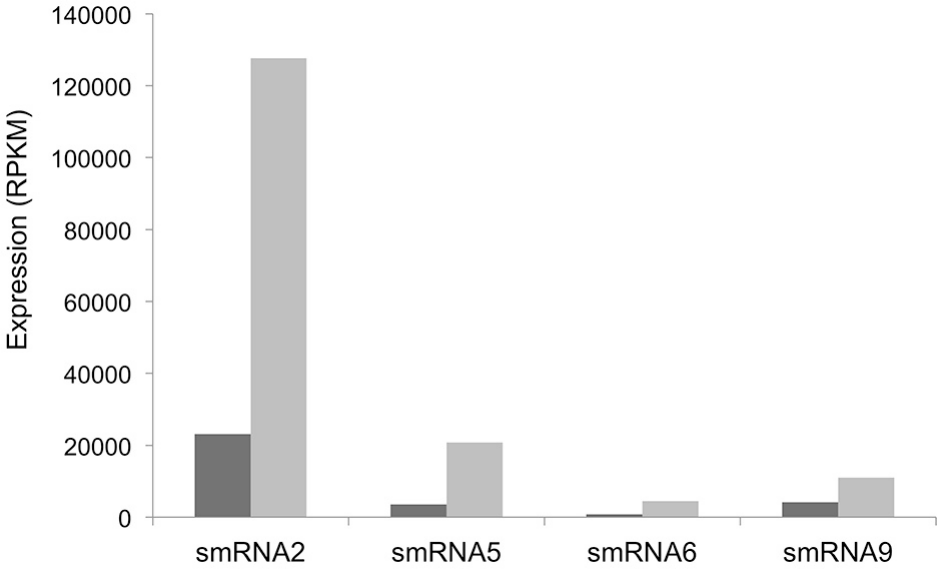
**Iron-mediated regulation of sRNAs.** The expression level in RPKM of each sRNA is shown on the y-axis. +Fe (dark gray bars): wild-type strain grown with 100 μM ferric nitrate for 60 min, −Fe (light gray bars): wild-type strain grown with 100 μM desferal for 60 min. All changes in expression were statistically significant with a *q*-value of <0.05.

Our RNA-seq analysis of sRNAs in *N. gonorrhoeae* utilized RNA isolated from the organism grown under high and low iron conditions. We next set out to determine which sRNAs respond to specific iron conditions. RNA from iron replete and deplete conditions was prepared and sequenced separately in these experiments allowing iron-mediated regulation of sRNAs to be observed. In addition, as these experiments were part of a larger study examining the global transcriptional response of *N. gonorrhoeae* to iron the entire transcriptome was sequenced without size selection. While fewer sRNAs overall were detected, all of the sRNAs identified and confirmed by Northern blot analysis above were also found under either iron replete or deplete conditions in these experiments and a subset were revealed to show varying abundance as a consequence of growth under variable iron conditions (Figure 5). Iron mediated regulation of sRNAs was observed with smRNAs 2, 5, 6, and 9 with all sRNAs being expressed more highly under low iron conditions. All of these changes were statistically significant with a *q*-value of <0.05.

**Figure 6:**
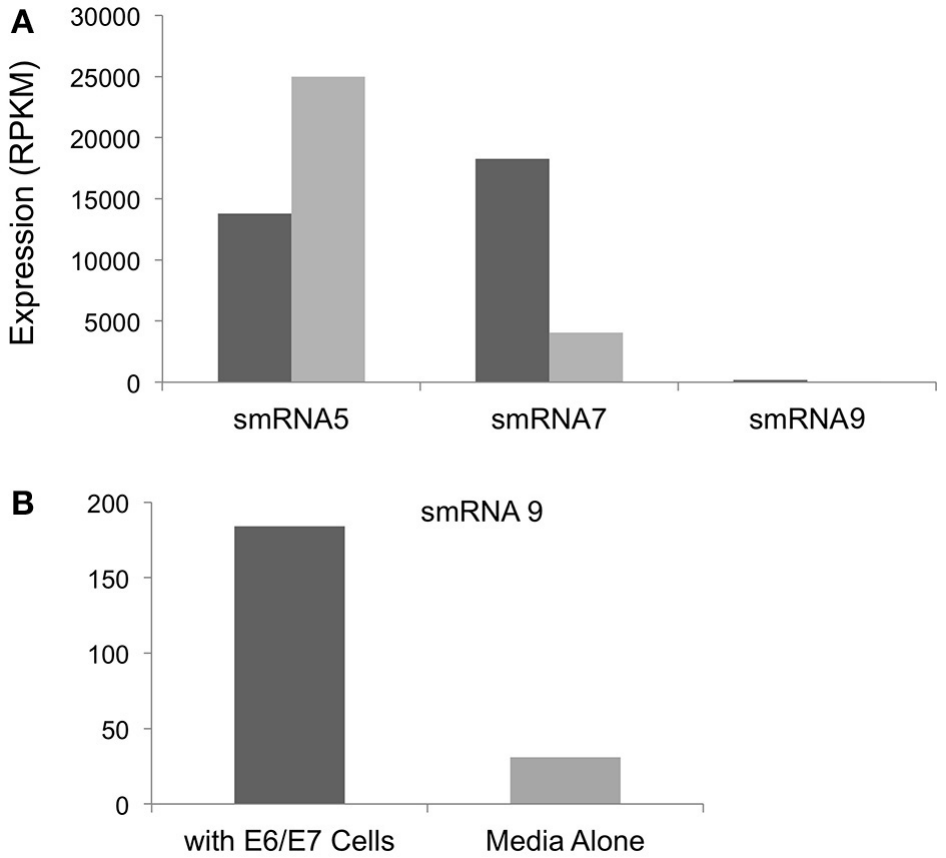
**Regulation of sRNAs during incubation with E6/E7 Endocervical cells. (A)** The expression level in RPKM of each sRNA shown on the y-axis. All changes in expression were statistically significant with a *q*-value of <0.05. Expression is shown during incubation with E6/E7 cells (dark gray bars) or with media alone (light gray bars). **(B)** smRNA 9 was expressed at significantly lower levels compared to smRNA 5 and 7 and is shown separately for ease of viewing.

In addition to iron we also examined expression of sRNAs during incubation with a transformed endocervical cell line. These cells have been used extensively by our group and others to study *N. gonorrhoeae* interactions with host cells. *N. gonorrhoeae* has been shown to replicate during co-incubation with these cells and to adhere and invade these cells in a relatively short time timeframe (>1 h) (Fichorova et al., 1997; Canny et al., 2006; Follows et al., 2009; Daou et al., 2013). Of the sRNAs that were examined by Northern blot analysis we did not detect smRNA 4 or 10 during incubation with epithelial cells or in media alone, likely due to the differences in culture conditions in our size selected data (iron replete or deplete) compared to growth in KSFM or with endocervical cells. However, all other sRNAs confirmed by Northern blot analysis were detected. Three such sRNAs showed changes in expression when we compared incubation with epithelial cells to growth in cell culture media alone. smRNAs 7 and 9 showed 4.4 and 5.9-fold increases in expression during incubation with epithelial cells compared to growth in media alone (Figure 6A). In contrast, smRNA 5 showed higher expression (1.8-fold) in media alone compared to incubation with epithelial cells (Figure 6B). As above, all of these changes were statistically significant with a *q*-value of <0.05.

### Additional sRNAs expressed under different conditions

In addition to the subset of seven sRNAs examined under all conditions (iron replete and deplete; incubation with or without endocervical cells) we also examined others sRNAs of *N. gonorrhoeae* under these conditions. We detected a total of 32 sRNAs larger than 30 nucleotides expressed under either iron replete or deplete conditions with 16 sRNAs showing at least 2-fold regulation when comparing iron replete and deplete conditions with a *q*-value of <0.1. Three sRNAs showed increased expression under iron replete conditions and 13 sRNAs showed decreased expression (Table S3). In contrast to iron regulation, experiments examining *N. gonorrhoeae* interaction with endocervical host cells involved more than one organism. Isolated RNA can be either from *N. gonorrhoeae* or from eukaryotic host cells. To focus only on the transcriptome of *N. gonorrhoeae* only RNA which aligned to the *N. gonorrhoeae* FA1090 genome was analyzed. When we analyzed RNA samples from *N. gonorrhoeae* grown in cell culture media alone we observed that a large proportion of RNA (75–80%) aligned to the *N. gonorrhoeae* genome.

In contrast, when we analyzed RNA samples obtained from *N. gonorrhoeae* incubated with endocervical cells only ~5% of RNA aligned to the *N. gonorrhoeae* genome. This is likely due to the large amount of eukaryotic RNA present in the system. We detected a total of 209 sRNAs greater than 30 nucleotides in length expressed either during incubation in cell culture media alone or with endocervical cells (Table S4). Of these, 131 showed differential expression of at least 2-fold with a *q*-value of <0.1 when examining incubation in cell culture media to *N. gonorrhoeae* incubated with endocervical cells. The vast majority of sRNA identified (129) showed increased expression when incubated with epithelial cells compared to media alone. Of the 32 sRNAs found to be expressed under iron replete or deplete conditions 11 (34%) were unique to those conditions and were not found when *N. gonorrhoeae* was incubated in cell culture media or with endocervical cells. This is despite the fact that more than 4.1-fold more sRNAs were detected under cell incubation conditions compared to iron conditions. Conversely, 188 sRNAs were detected only during incubation in cell culture media or with endocervical cells and not during growth in CDM under iron replete or deplete conditions. These results show that there is large variability in expression patterns of sRNAs in *N. gonorrhoeae*, however the large number of variables between broth culture and growth in cell culture media or with endocervical cells preclude a definitive identification of environmental conditions stimulating expression of these sRNAs.

When examining each of the four conditions individually there were also major differences in the expression patterns of sRNAs. Analysis of *N. gonorrhoeae* with endocervical cells detected 148 putative sRNAs and of these 99 were detected only during incubation with endocervical cells and under no other condition. Analysis of *N. gonorrhoeae* in media alone detected 49 putative sRNAs with only 1 being unique to this condition; there was significant overlap in sRNAs detected with endocervical cells compared to media alone. Growth of *N. gonorrhoeae* under iron replete conditions induced the expression of 18 sRNAs with 7 being unique to this condition. Growth of *N. gonorrhoeae* under iron deplete conditions induced expression of 15 sRNAs with 6 being unique to this condition (Table S5). These observations show that many sRNAs respond to very specific conditions present during growth and do not show constitutive expression. Such regulatory information will be of use when determining targets of sRNAs.

### Possible targets of identified sRNAs

sRNAs regulate mainly mRNA targets, a process carried out through homologous baseparing. To begin to define targets of the sRNA identified under the experimental conditions used in this study we utilized TargetRNA. We reasoned that if a given sRNA/mRNA pair were predicted to form a homologous base paring interaction and both were regulated under the same conditions the possibility exists that the sRNA could function as a regulator of the mRNA. All identified sRNAs showing regulation under at least one condition were examined and several possible targets were found for smRNA 9. Transcripts encoding NGO0825 (putative ferredoxin), NGO1183 (protein synthesis), NGO1439 (ATP-binding transporter), and *pykA* (pyruvate kinase) were all shown to contain regions of homology to smRNA 9 and to be regulated under the same conditions. NGO0825, NGO1183, and NGO1439 were all expressed more highly during incubation with *N. gonorrhoeae*. This regulatory pattern is identical to smRNA 9 and may suggest that smRNA 9 positive regulates these transcripts. In contrast, *pykA* exhibited greater expression during incubation with media alone, a regulatory pattern opposite that of smRNA 9, suggesting that smRNA 9 may negatively regulate this transcript.

## Discussion

Bacterial gene regulation via sRNAs is a field of rapidly advancing discovery. New technologies such as global RNA sequencing are making the discovery of novel sRNAs easier, as well as providing the first glimpses of the global sRNAome of bacteria where little is known about sRNA regulation, such as in the pathogen *N. gonorrhoeae*. It is possible that sRNAs may play a more prominent role in gene regulation in *N. gonorrhoeae* as compared to other bacteria due to the relatively low number of global proteomic transcription factors in the gonococcus. Despite this possibility as well as the analysis of more than 100 sRNAs in other organisms such as *E. coli*, there are only a handful of sRNAs that have been well characterized in *N. gonorrhoeae*. The analysis presented here sought to identify and characterize sRNAs more broadly in the gonococcus. RNA-seq analysis in *N. gonorrhoeae* identified several hundred instances of transcription that were outside or antisense to known genes. Interestingly, when using different experimental conditions, most notably a lack of RNA size selection and the inclusion of rRNA depletion, far fewer sRNAs were found. This may be of interest to other investigators focusing on identifying and analyzing classes of RNAs that are of relatively homogenous size. A gel electrophoresis step may increase the number of RNA transcripts detected and possibly allow the detection of transcripts expressed at very low levels. Of the 10 sRNAs that we chose from the 232 that were expressed under all conditions, the expression of seven was confirmed via Northern blot analysis and all seven had a size in the range of known sRNAs. While a number of putative sRNAs detected may represent 5′ or 3′ UTRs, due to the fact that 7/10 of the randomly tested sRNAs fulfill characteristics of sRNAs, it is reasonable to assume that a large number of the remaining 222 candidates indeed correspond to sRNAs. These results greatly increase the number of known sRNAs in the gonococcus and suggest that sRNA mediated gene regulation may be a commonly used mechanism for control of gene expression in the pathogenic *Neisseria*. Several of the sRNAs tested had a predicted RIT, yet the presence of an RIT was absent in the majority of sRNAs. We would therefore suggest that the presence or absence of a predicted RIT should not be considered a disqualifying characteristic in future analyses which focus on sRNAs in *Neisseria* and perhaps in other bacteria as well.

A subset of sRNAs were found to be antisense to known protein coding genes, such as smRNA 8 and 9. The increasing amount of anti-sense transcription being found in prokaryotic organisms has altered paradigms regarding genetic structure in these organisms. A study of *H. pylori* found that 50% of genes contained an anti-sense transcript on the opposite strand (Sharma et al., 2010). Our sRNA RNA-seq experiments showed that 21% (59/281) of the transcripts were expressed opposite to a known gene. When analyzing sRNAs, such transcripts are especially interesting given their strong potential for being post-transcriptional regulators. sRNAs function through base-pairing with mRNA targets, therefore such anti-sense transcripts may suggest a *cis*-regulatory role for the sRNA. Genomic location may also provide preliminary information regarding the role of individual sRNAs. In other bacteria, as well as *N. gonorrhoeae*, some sRNAs have been found to be in the vicinity of their target genes (Cahoon and Seifert, 2013). One of the sRNAs confirmed by Northern blot analysis in this study (smRNA 7) was found within the NgΦ1 bacteriophage genomic region previously identified in *N. gonorrhoeae* (Piekarowicz et al., 2007). Our laboratory, and others, has determined that phage DNA can have a role in regulatory pathways of *N. gonorrhoeae*. A mutant strain of *N. gonorrhoeae* lacking a phage associated repressor was found exhibit increased adherence and survival within epithelial cells as well as being more fit within a mouse model of infection as compared to the wild-type strain (Daou et al., 2013). It is possible that smRNA 7 as well as other sRNAs found in phage regions may have originally been part of the phage transcriptome but have now acquired new roles in regulatory pathways of *N. gonorrhoeae*. Another sRNA (coordinates 1327669-1327704) was also found in the *mtrCDE* locus, which encodes for an efflux pump in *N. gonorrhoeae*. We propose that searches for putative targets of these sRNAs should begin in these regions and that stimuli that regulate genes in these loci may also regulate these putative sRNAs.

When we examined the expression of sRNA during incubation of *N. gonorrhoeae* with human endocervical cells a much larger repertoire of sRNAs was identified despite the fact that input RNA for sequencing was not size selected. This may reflect that fact that regulation by sRNAs is an integral component of successful infection by *N. gonorrhoeae*. As a strict human pathogen the most commonly encountered set of stimuli for this organism involves human cells. Therefore, it is not surprising that a majority of regulatory pathways would respond to such stimuli. Interestingly, the overwhelming majority of these sRNAs showed increased expression during incubation with endocervical cells compared to media alone.

In summary, we report on the first RNA-sequencing study of the human pathogen *N. gonorrhoeae* specifically focused on identification of novel sRNAs. We discovered several hundred putative sRNAs and confirm the expression of seven new sRNAs in the gonococcus. It is likely that several of these transcripts act as post-transcriptional regulators and targets of these sRNAs are currently being characterized. The gonococcus contains comparatively few protein regulators and as such may be more likely to use sRNA-mediated mechanisms of control to regulate gene expression. This observation, combined with the fact that many sRNAs known in other organisms are involved in pathogenesis, emphasize the need for continued study of sRNAs in this organism. These experiments begin the work of characterizing sRNAs of *N. gonorrhoeae* as well as how they may be regulated and open up new avenues of genetic regulatory mechanisms in this human pathogen.

### Conflict of interest statement

The authors declare that the research was conducted in the absence of any commercial or financial relationships that could be construed as a potential conflict of interest.

## Supplementary material

Figure S1 **Primer extension analysis of identified sRNAs**. Between 8 and 10 μg of RNA isolated from an iron replete growth condition was incubated with a radiolabeled DNA oligonucleotide that was homologous to the RNA sequence under analysis. Reverse transcription was allowed to take place and products were analyzed on an 8% TBE-Urea polyacrylamide gel. Radiolabeled size markers are shown and sizes of individual markers indicated. Samples were also examined without input RNA as a negative control as shown with a (-) sign. Experiments were preformed three times and a representative film is shown. Probes used: smRNA 7-CAGATATATTCGGACTGCACCTC, smRNA8-GAATGTGTGCCAAGTCTACA.
