# Supplementary figures and images for: Identification of sRNAs expressed by the human pathogen Neisseria gonorrhoeae under disparate growth conditions
Source: Front Microbiol. 2014 Aug 28;5:456. doi: 10.3389/fmicb.2014.00456 (PMC4148029; doi:10.3389/fmicb.2014.00456)

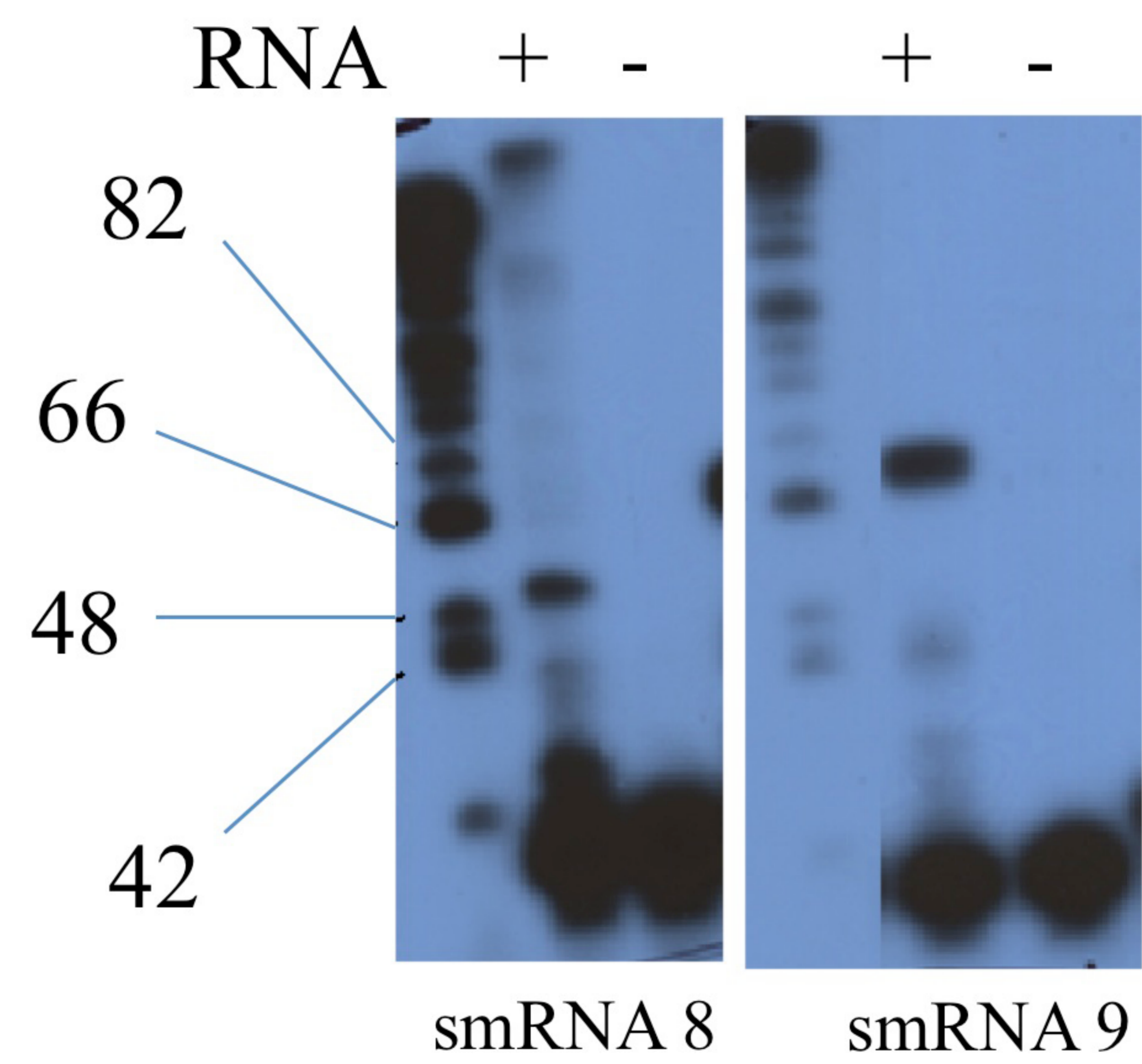

Supplement: Figure S1 — Primer extension analysis of identified sRNAs. Between 8 and 10 μg of RNA isolated from an iron replete growth condition was incubated with a radiolabeled DNA oligonucleotide that was homologous to the RNA sequence under analysis. Reverse transcription was allowed to take place and products were analyzed on an 8% TBE-Urea polyacrylamide gel. Radiolabeled size markers are shown and sizes of individual markers indicated. Samples were also examined without input RNA as a negative control as shown with a (-) sign. Experiments were preformed three times and a representative film is shown. Probes used: smRNA 7-CAGATATATTCGGACTGCACCTC, smRNA8-GAATGTGTGCCAAGTCTACA. [file Presentation1.PDF]
