# Supplementary material for: Identification of sRNAs expressed by the human pathogen Neisseria gonorrhoeae under disparate growth conditions
Source: Front Microbiol. 2014 Aug 28;5:456. doi: 10.3389/fmicb.2014.00456 (PMC4148029; doi:10.3389/fmicb.2014.00456)
Supplement: Supplementary file 2 [file DataSheet1.DOCX]

**Table S1**

| **Name** | **Northern Probe** |
| --- | --- |
| smRNA1 | ATGCCTGGTAAATTGCCAATCTTCTACGGTGTTGCATATTGACCCTTTC |
| smRNA2 | CAAACAGGCTGTGCTTATTCCCTGTTGAACAGGTATTGTATTCGCAGCCC |
| smRNA3 | TTGTAAACGTTTTGCCTTGCATGATGAAATGCCGTCCGAAGATAAAAATATT |
| smRNA4 | AATTCAGTGTTTTACGGGAATCTGAGACCCCGGTATGCCCGCATCTGCTT |
| smRNA5 | CTGACCCGGTGTTCCGATTTGCCATGCGGGGAGACCCGCAAC |
| smRNA6 | GGCAACTTGCTTTCATTACTTGACCGCGTATCAACTACGGGAGCCTCTG |
| smRNA7 | CCGAATATATCTGCCTGCTGTTTCCTCTTTATTCAGCCTTTATAATA |
| smRNA8 | GTCTACAAAGGAGAAGTAGAGGAGAAAAATCAAGCTGCATCAAGCAAC |
| smRNA9 | ACTATTAACTGACTACTCGAACCAGCTAAC |
| smRNA10 | ACCTTTTCACCCTTGCCTGTGCTGCCAAAGCAGCCATCGGCGGTTTTGCT |

**Table S2**

| **Transcription Start^*^** | **Transcription Stop^*^** | **Strand^#^** | **Length** | **Product^\|\|^** | **Expression Level**^§^ |
| --- | --- | --- | --- | --- | --- |
| 11980 | 12055 | + | 76 | - | 1157 |
| 17436 | 17491 | + | 56 | antisense: NGO0021 | 1747 |
| 18047 | 18081 | + | 35 | antisense: NGO0021 | 383 |
| 19676 | 19637 | - | 40 | - | 750 |
| 36109 | 36075 | - | 35 | antisense: NGO0039 | 889 |
| 38028 | 38084 | + | 57 | - | 1154 |
| 39872 | 39901 | + | 30 | antisense: prmA | 338 |
| 48350 | 48242 | - | 109 | - | 1573 |
| 48458 | 48421 | - | 38 | - | 462 |
| 48871 | 48798 | - | 74 | - | 1546 |
| 49020 | 48983 | - | 38 | - | 470 |
| 49474 | 49366 | - | 109 | - | 1004 |
| 55800 | 55699 | - | 102 | - | 748 |
| 56005 | 55896 | - | 110 | - | 2822 |
| 56262 | 56203 | - | 60 | - | 1051 |
| 56439 | 56411 | - | 29 | - | 345 |
| 56783 | 56762 | - | 22 | - | 363 |
| 68600 | 68484 | - | 117 | - | 601 |
| 68928 | 68798 | - | 131 | - | 1652 |
| 69189 | 69178 | - | 12 | - | 648 |
| 69345 | 69295 | - | 51 | - | 696 |
| 69617 | 69565 | - | 53 | - | 2051 |
| 69757 | 69728 | - | 30 | - | 404 |
| 69877 | 69859 | - | 19 | - | 576 |
| 74178 | 74142 | - | 37 | - | 724 |
| 74342 | 74303 | - | 40 | - | 1028 |
| 74690 | 74627 | - | 64 | - | 1891 |
| 75828 | 75790 | - | 39 | - | 715 |
| 78404 | 78381 | - | 24 | - | 312 |
| 78553 | 78594 | + | 42 | - | 653 |
| 81175 | 81137 | - | 39 | antisense: NGO0077 | 297 |
| 81512 | 81479 | - | 34 | - | 812 |
| 97215 | 97181 | - | 35 | - | 445 |
| 100253 | 100214 | - | 40 | - | 920 |
| 134524 | 134485 | - | 40 | antisense: NGO0123 | 811 |
| 155290 | 155328 | + | 39 | - | 623 |
| 156265 | 156211 | - | 55 | - | 3464 |
| 166853 | 166890 | + | 38 | - | 482 |
| 174021 | 174045 | + | 25 | - | 521 |
| 187718 | 187676 | - | 43 | - | 679 |
| 207901 | 207953 | + | 53 | - | 3372 |
| 243129 | 243168 | + | 40 | - | 536 |
| 251359 | 251411 | + | 53 | - | 2972 |
| 260471 | 260510 | + | 40 | - | 1072 |
| 300248 | 300274 | + | 27 | - | 421 |
| 324576 | 324615 | + | 40 | antisense: NGO0327 | 919 |
| 326623 | 326663 | + | 41 | antisense: NGO0330 | 2136 |
| 326722 | 326753 | + | 32 | antisense: NGO0330 | 695 |
| 327457 | 327414 | - | 44 | - | 584 |
| 336903 | 336944 | + | 42 | - | 571 |
| 339014 | 339053 | + | 40 | antisense: NGO0344 | 1376 |
| 393472 | 393515 | + | 44 | - | 1893 |
| 411782 | 411800 | + | 19 | - | 492 |
| 411921 | 411960 | + | 40 | - | 1301 |
| 425599 | 425638 | + | 40 | - | 746 |
| 425724 | 425710 | - | 15 | - | 547 |
| 431685 | 431714 | + | 30 | - | 475 |
| 431889 | 431921 | + | 33 | - | 308 |
| 432025 | 432064 | + | 40 | - | 741 |
| 432169 | 432274 | + | 106 | - | 450 |
| 432829 | 432935 | + | 107 | - | 538 |
| 433286 | 433324 | + | 39 | - | 550 |
| 443849 | 443892 | + | 44 | - | 1328 |
| 446988 | 447108 | + | 121 | - | 4100 |
| 471809 | 471771 | - | 39 | - | 658 |
| 471816 | 471905 | + | 90 | - | 21216 |
| 483127 | 483088 | - | 40 | antisense: NGO0506 | 607 |
| 483990 | 483904 | - | 87 | antisense: NGO0507 | 918 |
| 484258 | 484242 | - | 17 | antisense: NGO0508 | 548 |
| 497896 | 497913 | + | 18 | antisense: NGO0524 | 589 |
| 498147 | 498230 | + | 84 | - | 873 |
| 511229 | 511271 | + | 43 | - | 1945 |
| 512083 | 512136 | + | 54 | - | 969 |
| 514122 | 514177 | + | 56 | - | 901 |
| 514944 | 514979 | + | 36 | - | 678 |
| 546118 | 546087 | - | 32 | - | 551 |
| 566429 | 566468 | + | 40 | antisense: NGO0579 | 550 |
| 571077 | 571145 | + | 69 | - | 530 |
| 571208 | 571274 | + | 67 | - | 1847 |
| 578155 | 578114 | - | 42 | - | 679 |
| 584570 | 584554 | - | 17 | - | 538 |
| 588288 | 588249 | - | 40 | - | 601 |
| 599406 | 599444 | + | 39 | - | 493 |
| 634295 | 634332 | + | 38 | antisense: rbfA | 527 |
| 637363 | 637414 | + | 52 | - | 16150 |
| 640902 | 640863 | - | 40 | antisense: NGO0651 | 726 |
| 645629 | 645668 | + | 40 | - | 793 |
| 655058 | 655071 | + | 14 | - | 570 |
| 669002 | 669036 | + | 35 | - | 393 |
| 682928 | 682889 | - | 40 | - | 621 |
| 688409 | 688398 | - | 12 | - | 434 |
| 699366 | 699321 | - | 46 | - | 794 |
| 714908 | 714867 | - | 42 | - | 794 |
| 738786 | 738753 | - | 34 | - | 288 |
| 745976 | 746015 | + | 40 | - | 1287 |
| 755847 | 755886 | + | 40 | antisense: ruvB | 459 |
| 797592 | 797643 | + | 52 | - | 1178 |
| 833494 | 833448 | - | 47 | - | 1784 |
| 841253 | 841206 | - | 48 | - | 2462 |
| 843096 | 843058 | - | 39 | antisense: NGO0859 | 848 |
| 846376 | 846332 | - | 45 | - | 3021 |
| 846573 | 846620 | + | 48 | - | 1936 |
| 846713 | 846797 | + | 85 | - | 691 |
| 847401 | 847383 | - | 19 | - | 456 |
| 847404 | 847498 | + | 95 | - | 1265 |
| 847838 | 847853 | + | 16 | - | 528 |
| 848409 | 848437 | + | 29 | - | 530 |
| 862942 | 863067 | + | 126 | - | 3497 |
| 863131 | 863310 | + | 180 | - | 24422 |
| 870849 | 870808 | - | 42 | antisense: NGO0892 | 3707 |
| 876298 | 876330 | + | 33 | - | 424 |
| 918967 | 919005 | + | 39 | - | 561 |
| 921101 | 921061 | - | 41 | - | 1610 |
| 925187 | 925314 | + | 128 | - | 607 |
| 939809 | 939775 | - | 35 | antisense: NGO0967 | 617 |
| 946696 | 946739 | + | 44 | - | 2157 |
| 954932 | 954971 | + | 40 | - | 1376 |
| 986196 | 986157 | - | 40 | - | 717 |
| 998885 | 998513 | - | 373 | - | 828 |
| 999370 | 999420 | + | 51 | antisense: NGO1037 | 937 |
| 999958 | 999913 | - | 46 | - | 1182 |
| 1000324 | 1000292 | - | 33 | - | 760 |
| 1000861 | 1000805 | - | 57 | - | 1442 |
| 1032157 | 1032174 | + | 18 | - | 593 |
| 1032410 | 1032421 | + | 12 | - | 603 |
| 1032956 | 1033037 | + | 82 | - | 830 |
| 1033417 | 1033449 | + | 33 | - | 491 |
| 1034087 | 1034113 | + | 27 | - | 539 |
| 1034997 | 1035019 | + | 23 | - | 504 |
| 1035402 | 1035466 | + | 65 | - | 2164 |
| 1035800 | 1035845 | + | 46 | - | 1183 |
| 1051765 | 1051802 | + | 38 | antisense: NGO1092 | 709 |
| 1072229 | 1072272 | + | 44 | - | 1004 |
| 1082522 | 1082560 | + | 39 | antisense: NGO1138 | 577 |
| 1100132 | 1100093 | - | 40 | - | 547 |
| 1105232 | 1105270 | + | 39 | antisense: NGO1165 | 555 |
| 1108221 | 1108179 | - | 43 | - | 1053 |
| 1113497 | 1113469 | - | 29 | - | 471 |
| 1122708 | 1122779 | + | 72 | - | 2014 |
| 1122880 | 1122930 | + | 51 | - | 461 |
| 1123029 | 1123068 | + | 40 | - | 1023 |
| 1130183 | 1130142 | - | 42 | antisense: NGO1187 | 1854 |
| 1133757 | 1133797 | + | 41 | - | 1129 |
| 1149059 | 1149100 | + | 42 | - | 667 |
| 1157235 | 1157196 | - | 40 | antisense: uvrA | 512 |
| 1172605 | 1172644 | + | 40 | - | 608 |
| 1172616 | 1172577 | - | 40 | - | 1404 |
| 1209225 | 1209186 | - | 40 | - | 1562 |
| 1215531 | 1215569 | + | 39 | - | 456 |
| 1218182 | 1218221 | + | 40 | antisense: NGO1263 | 632 |
| 1231248 | 1231298 | + | 51 | - | 2268 |
| 1231713 | 1231873 | + | 161 | - | 1284 |
| 1232131 | 1232195 | + | 65 | - | 805 |
| 1233810 | 1233771 | - | 40 | - | 601 |
| 1241480 | 1241442 | - | 39 | - | 582 |
| 1248708 | 1248738 | + | 31 | antisense: NGO1293 | 454 |
| 1248894 | 1248928 | + | 35 | - | 499 |
| 1249335 | 1249372 | + | 38 | antisense: NGO1294 | 601 |
| 1264761 | 1264738 | - | 24 | antisense: NGO1307 | 523 |
| 1281321 | 1281332 | + | 12 | - | 509 |
| 1284789 | 1284828 | + | 40 | - | 1242 |
| 1316950 | 1316928 | - | 23 | - | 333 |
| 1327704 | 1327669 | - | 36 | - | 832 |
| 1339136 | 1339173 | + | 38 | antisense: NGO1374 | 548 |
| 1342188 | 1342131 | - | 58 | - | 2908 |
| 1376054 | 1376028 | - | 27 | antisense: NGO1411 | 492 |
| 1384187 | 1384171 | - | 17 | antisense: NGO1420 | 532 |
| 1386475 | 1386443 | - | 33 | - | 541 |
| 1388431 | 1388275 | - | 157 | - | 934 |
| 1388603 | 1388594 | - | 10 | - | 564 |
| 1389153 | 1389122 | - | 32 | - | 516 |
| 1425672 | 1425660 | - | 13 | antisense: NGO1458 | 346 |
| 1425954 | 1425936 | - | 19 | antisense: NGO1459 | 369 |
| 1426231 | 1426215 | - | 17 | - | 356 |
| 1427812 | 1427744 | - | 69 | - | 1811 |
| 1428100 | 1428155 | + | 56 | - | 1021 |
| 1428234 | 1428182 | - | 53 | - | 2087 |
| 1433219 | 1433246 | + | 28 | - | 548 |
| 1443167 | 1443129 | - | 39 | - | 582 |
| 1443425 | 1443365 | - | 61 | - | 415 |
| 1446395 | 1446356 | - | 40 | - | 1470 |
| 1459030 | 1458991 | - | 40 | - | 854 |
| 1468604 | 1468635 | + | 32 | - | 470 |
| 1468853 | 1468814 | - | 40 | - | 701 |
| 1481801 | 1481744 | - | 58 | antisense: NGO1513 | 1642 |
| 1482394 | 1482425 | + | 32 | - | 592 |
| 1490707 | 1490668 | - | 40 | - | 1518 |
| 1510500 | 1510453 | - | 48 | - | 1927 |
| 1530374 | 1530413 | + | 40 | - | 627 |
| 1531130 | 1531177 | + | 48 | - | 765 |
| 1531525 | 1531652 | + | 128 | - | 610 |
| 1540599 | 1540572 | - | 28 | antisense: NGO1568 | 378 |
| 1541328 | 1541287 | - | 42 | - | 693 |
| 1541479 | 1541492 | + | 14 | - | 303 |
| 1543045 | 1543085 | + | 41 | - | 798 |
| 1557931 | 1557918 | - | 14 | antisense: NGO1582 | 534 |
| 1562400 | 1562439 | + | 40 | - | 485 |
| 1562609 | 1562649 | + | 41 | - | 1066 |
| 1562769 | 1562731 | - | 39 | - | 523 |
| 1568895 | 1568941 | + | 47 | antisense: NGO1597 | 834 |
| 1569640 | 1569652 | + | 13 | antisense: NGO1597 | 595 |
| 1592980 | 1593008 | + | 29 | antisense: NGO1629 | 541 |
| 1601595 | 1601633 | + | 39 | antisense: NGO1643 | 590 |
| 1609960 | 1610059 | + | 100 | - | 616 |
| 1617089 | 1617023 | - | 67 | - | 1048 |
| 1617217 | 1617188 | - | 30 | - | 692 |
| 1624114 | 1624155 | + | 42 | - | 3100 |
| 1632423 | 1632385 | - | 39 | antisense: NGO1678 | 521 |
| 1638514 | 1638467 | - | 48 | - | 1983 |
| 1642857 | 1642818 | - | 40 | - | 570 |
| 1672527 | 1672503 | - | 25 | - | 371 |
| 1712642 | 1712588 | - | 55 | - | 1047 |
| 1714045 | 1714084 | + | 40 | antisense: NGO1752 | 711 |
| 1726101 | 1726141 | + | 41 | - | 924 |
| 1726837 | 1726903 | + | 67 | - | 1248 |
| 1727831 | 1727868 | + | 38 | - | 641 |
| 1729660 | 1729699 | + | 40 | - | 885 |
| 1742684 | 1742715 | + | 32 | - | 384 |
| 1750935 | 1750897 | - | 39 | - | 536 |
| 1763021 | 1762953 | - | 69 | - | 578 |
| 1779932 | 1779970 | + | 39 | - | 663 |
| 1786704 | 1786744 | + | 41 | - | 944 |
| 1788241 | 1788288 | + | 48 | - | 1045 |
| 1789890 | 1789856 | - | 35 | - | 502 |
| 1830400 | 1830335 | - | 66 | - | 608 |
| 1830647 | 1830795 | + | 149 | - | 1116 |
| 1831312 | 1831351 | + | 40 | - | 791 |
| 1843127 | 1843195 | + | 69 | antisense: NGO1872 | 2830 |
| 1844945 | 1844891 | - | 55 | - | 1009 |
| 1847298 | 1847205 | - | 94 | - | 610 |
| 1848428 | 1848301 | - | 128 | - | 1595 |
| 1848522 | 1848483 | - | 40 | - | 909 |
| 1848649 | 1848610 | - | 40 | - | 919 |
| 1849639 | 1849680 | + | 42 | - | 1100 |
| 1850772 | 1850742 | - | 31 | - | 510 |
| 1850894 | 1850856 | - | 39 | - | 680 |
| 1871765 | 1871806 | + | 42 | - | 666 |
| 1887488 | 1887449 | - | 40 | - | 795 |
| 1887777 | 1887767 | - | 11 | - | 567 |
| 1888160 | 1888128 | - | 33 | - | 544 |
| 1890399 | 1890349 | - | 51 | - | 1090 |
| 1890986 | 1890976 | - | 11 | - | 463 |
| 1910179 | 1910217 | + | 39 | - | 808 |
| 1910325 | 1910411 | + | 87 | - | 610 |
| 1914339 | 1914403 | + | 65 | - | 748 |
| 1914663 | 1914701 | + | 39 | - | 698 |
| 1922100 | 1922137 | + | 38 | - | 439 |
| 1924430 | 1924767 | + | 338 | antisense: NGO1952 | 13282 |
| 1927036 | 1927001 | - | 36 | - | 661 |
| 1929631 | 1929670 | + | 40 | antisense: NGO1955 | 1578 |
| 1929801 | 1929856 | + | 56 | antisense: NGO1955 | 1300 |
| 1930001 | 1930151 | + | 151 | antisense: NGO1955 | 696 |
| 1930981 | 1931045 | + | 65 | antisense: NGO1955 | 623 |
| 1935091 | 1935129 | + | 39 | - | 733 |
| 1941314 | 1941350 | + | 37 | - | 700 |
| 1941695 | 1941705 | + | 11 | - | 497 |
| 1942114 | 1942209 | + | 96 | - | 333 |
| 1944232 | 1944200 | - | 33 | - | 801 |
| 1947140 | 1947117 | - | 24 | - | 718 |
| 1950423 | 1950461 | + | 39 | - | 539 |
| 1962309 | 1962272 | - | 38 | antisense: NGO1989 | 527 |
| 1976122 | 1976057 | - | 66 | - | 864 |
| 1989742 | 1989785 | + | 44 | antisense: NGO2019 | 2736 |
| 2006699 | 2006752 | + | 54 | - | 873 |
| 2008448 | 2008415 | - | 34 | antisense: NGO2038 | 523 |
| 2022362 | 2022335 | - | 28 | - | 367 |
| 2036974 | 2036922 | - | 53 | - | 2040 |
| 2039011 | 2038925 | - | 87 | - | 342 |
| 2065298 | 2065338 | + | 41 | antisense: NGO2088 | 1803 |
| 2065815 | 2065857 | + | 43 | antisense: NGO2088 | 950 |
| 2066055 | 2066096 | + | 42 | antisense: NGO2089 | 701 |
| 2084650 | 2084666 | + | 17 | - | 520 |
| 2091565 | 2091601 | + | 37 | - | 595 |
| 2091707 | 2091716 | + | 10 | - | 331 |
| 2109261 | 2109300 | + | 40 | - | 400 |
| 2111961 | 2112000 | + | 40 | - | 756 |
| 2116553 | 2116586 | + | 34 | - | 652 |
| 2120891 | 2120923 | + | 33 | antisense: NGO2142 | 837 |
| 2131887 | 2131927 | + | 41 | - | 971 |
| 2142367 | 2142406 | + | 40 | antisense: NGO2165 | 669 |

*: Transcription start and stop sites are shown according to the Neisseria gonorrhoeae FA1090

#: Whether the putative sRNA is expressed from the coding (+) or complement (-) strand

||: Putative sRNAs that are antisense to known ORFs are indicated

§: The expression level of each putative sRNA in RPKM is shown

**Table S3**

| **Transcription Start^*^** | **Transcription Stop^*^** | **Strand^#^** | **Expression + Fe^&^** | **Expression – Fe^&^** | **qValue** | **Fold Change: WT+/WT-** |
| --- | --- | --- | --- | --- | --- | --- |
| 69641 | 69550 | - | 2140 | 1397 | 0.758231076 | 1.531853973 |
| 69749 | 69718 | - | 1005 | 2528 | 3.81E-08 | 0.397547468 |
| 326678 | 326689 | + | 157 | 1274 | 3.20E-26 | 0.123233909 |
| 471834 | 471942 | + | 23144 | 127641 | 0.019764547 | 0.181321049 |
| 591088 | 591029 | - | 1501 | 888 | 1 | 1.690315315 |
| 637374 | 637441 | + | 2067 | 1632 | 0.303533658 | 1.266544118 |
| 785861 | 785897 | + | 2663 | 66 | 0 | 40.34848485 |
| 862950 | 863329 | + | 32234 | 40647 | 0.856938336 | 0.793022855 |
| 870846 | 870744 | - | 3577 | 20787 | 3.86E-52 | 0.172078703 |
| 894770 | 894731 | - | 491 | 3475 | 1.43E-75 | 0.141294964 |
| 965198 | 965179 | - | 1248 | 523 | 0.327571652 | 2.38623327 |
| 1035409 | 1035469 | + | 2784 | 2176 | 0.334193337 | 1.279411765 |
| 1035826 | 1035860 | + | 1024 | 766 | 0.980587961 | 1.336814621 |
| 1051765 | 1051794 | + | 834 | 4470 | 1.42E-44 | 0.186577181 |
| 1090184 | 1090129 | - | 1908 | 1207 | 1 | 1.58077879 |
| 1116048 | 1116079 | + | 1294 | 2553 | 1.65E-05 | 0.506854681 |
| 1231709 | 1231950 | + | 2169 | 1331 | 1 | 1.629601803 |
| 1248768 | 1248820 | + | 1828 | 863 | 0.489576432 | 2.118192352 |
| 1248952 | 1249035 | + | 4147 | 10968 | 2.07E-08 | 0.378099927 |
| 1258149 | 1258184 | + | 1283 | 2607 | 8.91E-07 | 0.492136555 |
| 1372541 | 1372494 | - | 1757 | 1605 | 0.269475208 | 1.09470405 |
| 1428226 | 1428167 | - | 2018 | 1596 | 0.367969594 | 1.264411028 |
| 1443403 | 1443363 | - | 1750 | 1263 | 0.786669252 | 1.385589865 |
| 1531309 | 1531359 | + | 1797 | 809 | 0.391717394 | 2.221260816 |
| 1608627 | 1608713 | + | 2383 | 4804 | 1.79E-07 | 0.496044963 |
| 1631058 | 1630998 | - | 2848 | 1508 | 1 | 1.888594164 |
| 1631171 | 1631195 | + | 2710 | 713 | 0.001152477 | 3.800841515 |
| 1638473 | 1638448 | - | 305 | 2686 | 9.15E-72 | 0.11355175 |
| 1643726 | 1643646 | - | 2214 | 1403 | 0.768300331 | 1.578047042 |
| 1724648 | 1724690 | + | 1611 | 4286 | 4.06E-15 | 0.375874942 |
| 1796159 | 1796046 | - | 3665 | 1880 | 0.733965511 | 1.949468085 |
| 1808892 | 1808878 | - | 1803 | 1375 | 0.980587961 | 1.311272727 |
| 1823627 | 1823617 | - | 1468 | 618 | 0.401796758 | 2.375404531 |
| 1824955 | 1824934 | - | 2671 | 1712 | 1 | 1.560163551 |
| 1830718 | 1830788 | + | 682 | 1796 | 7.08E-13 | 0.379732739 |
| 1872877 | 1872914 | + | 1321 | 2611 | 5.56E-07 | 0.505936423 |
| 1910182 | 1910246 | + | 1565 | 879 | 1 | 1.780432309 |
| 1924431 | 1924815 | + | 21215 | 40246 | 0.927420866 | 0.527133131 |
| 1976094 | 1976004 | - | 57 | 10661 | 0 | 0.00534659 |
| 1981301 | 1981231 | - | 1441 | 343 | 2.96E-05 | 4.201166181 |
| 2030550 | 2030573 | + | 1733 | 1365 | 0.764114699 | 1.26959707 |
| 2036964 | 2036907 | - | 1899 | 1648 | 0.232515233 | 1.152305825 |
| 2139801 | 2139746 | - | 1697 | 973 | 1 | 1.744090442 |

*: Transcription start and stop sites are shown according to the Neisseria gonorrhoeae FA1090

#: Whether the putative sRNA is expressed from the coding (+) or complement (-) strand

&: The expression level of each putative sRNA in RPKM is shown under iron replete conditions (+Fe) or iron deplete conditions (-Fe).

**Table S4**

| **Transcription Start^*^** | **Transcription Stop^*^** | **Strand^#^** | **Expression with E6/E7 cells^&^** | **Expression Media only^&^** | **qValue** | **Fold Change: E6 cells/Media alone** |
| --- | --- | --- | --- | --- | --- | --- |
| 52360 | 52327 | - | 158 | 26 | 4.07E-13 | 6.08 |
| 68347 | 68452 | + | 146 | 62 | 1 | 2.35 |
| 69335 | 69299 | - | 0 | 410 | 1 | 0.00 |
| 69610 | 69524 | - | 12 | 924 | 1 | 0.01 |
| 69762 | 69702 | - | 25 | 582 | 1 | 0.04 |
| 74220 | 74269 | + | 241 | 45 | 2.83E-15 | 5.36 |
| 75879 | 76037 | + | 324 | 93 | 2.65E-06 | 3.48 |
| 75934 | 75904 | - | 179 | 63 | 0.35496535 | 2.84 |
| 76120 | 75994 | - | 342 | 40 | 1.50E-71 | 8.55 |
| 81518 | 81474 | - | 511 | 116 | 8.09E-12 | 4.41 |
| 100020 | 100105 | + | 1726 | 113 | 0 | 15.27 |
| 102572 | 102533 | - | 273 | 63 | 1.95E-10 | 4.33 |
| 103150 | 103232 | + | 635 | 45 | 2.00E-225 | 14.11 |
| 108616 | 108576 | - | 352 | 91 | 2.61E-07 | 3.87 |
| 111925 | 111961 | + | 188 | 96 | 0.99782526 | 1.96 |
| 130444 | 130534 | + | 97 | 43 | 0.03957783 | 2.26 |
| 130801 | 130832 | + | 172 | 19 | 3.32E-32 | 9.05 |
| 137364 | 137325 | - | 209 | 75 | 0.04526293 | 2.79 |
| 164091 | 164041 | - | 1803 | 251 | 1.56E-44 | 7.18 |
| 167067 | 167171 | + | 0 | 350 | 1 | 0.00 |
| 204180 | 204141 | - | 129 | 61 | 0.08815452 | 2.11 |
| 207906 | 207860 | - | 1613 | 243 | 3.06E-36 | 6.64 |
| 227254 | 227305 | + | 1784 | 290 | 2.39E-32 | 6.15 |
| 248865 | 248914 | + | 359 | 140 | 2.30E-04 | 2.56 |
| 259160 | 259213 | + | 174 | 199 | 9.24E-08 | 0.87 |
| 260127 | 260159 | + | 3 | 106 | 1.75E-48 | 0.03 |
| 260249 | 260298 | + | 236 | 55 | 1.41E-08 | 4.29 |
| 260455 | 260514 | + | 1607 | 268 | 3.39E-30 | 6.00 |
| 260545 | 260513 | - | 142 | 13 | 1.90E-48 | 10.92 |
| 263443 | 263410 | - | 374 | 158 | 0.00889428 | 2.37 |
| 270189 | 270150 | - | 191 | 249 | 1 | 0.77 |
| 300246 | 300287 | + | 140 | 211 | 1 | 0.66 |
| 321747 | 321786 | + | 501 | 363 | 1 | 1.38 |
| 327453 | 327375 | - | 15 | 549 | 1 | 0.03 |
| 329666 | 329704 | + | 222 | 110 | 0.01261913 | 2.02 |
| 334670 | 334759 | + | 1351 | 457 | 3.47E-18 | 2.96 |
| 341477 | 341518 | + | 1032 | 432 | 2.22E-04 | 2.39 |
| 423972 | 423900 | - | 1198 | 198 | 3.25E-30 | 6.05 |
| 424933 | 424973 | + | 529 | 208 | 0.00596529 | 2.54 |
| 425730 | 425681 | - | 451 | 116 | 1.99E-07 | 3.89 |
| 441351 | 441298 | - | 997 | 425 | 0.05541008 | 2.35 |
| 443849 | 443953 | + | 99 | 47 | 0.19493768 | 2.11 |
| 445493 | 445523 | + | 185 | 158 | 1 | 1.17 |
| 446989 | 447070 | + | 144 | 64 | 0.32413875 | 2.25 |
| 453642 | 453761 | + | 122 | 49 | 0.12334806 | 2.49 |
| 471834 | 471928 | + | 0 | 9925 | 1 | 0.00 |
| 511220 | 511259 | + | 184 | 74 | 0.29858009 | 2.49 |
| 557370 | 557424 | + | 1600 | 279 | 1.52E-26 | 5.73 |
| 569297 | 569338 | + | 219 | 496 | 1 | 0.44 |
| 587453 | 587350 | - | 144 | 5 | 0 | 28.80 |
| 587760 | 587723 | - | 163 | 3 | 0 | 54.33 |
| 588287 | 588017 | - | 230 | 4 | 0 | 57.50 |
| 588804 | 588839 | + | 155 | 7 | 1.50E-228 | 22.14 |
| 599324 | 599363 | + | 215 | 74 | 0.00386464 | 2.91 |
| 600258 | 600311 | + | 1124 | 194 | 4.15E-27 | 5.79 |
| 621847 | 621886 | + | 294 | 216 | 1 | 1.36 |
| 637363 | 637414 | + | 8773 | 4676 | 0.18596074 | 1.88 |
| 639058 | 639120 | + | 1731 | 305 | 1.45E-28 | 5.68 |
| 661057 | 661093 | + | 151 | 85 | 0.11332373 | 1.78 |
| 711520 | 711571 | + | 1200 | 112 | 3.00E-129 | 10.71 |
| 745898 | 745954 | + | 851 | 364 | 0.16195909 | 2.34 |
| 823837 | 823782 | - | 1866 | 267 | 6.54E-44 | 6.99 |
| 827247 | 827284 | + | 188 | 22 | 3.32E-33 | 8.55 |
| 846724 | 846794 | + | 254 | 96 | 0.01190405 | 2.65 |
| 862950 | 863311 | + | 26488 | 15533 | 0.60808257 | 1.71 |
| 876297 | 876263 | - | 330 | 17 | 6.72E-200 | 19.41 |
| 876823 | 876784 | - | 199 | 45 | 1.55E-06 | 4.42 |
| 877177 | 877123 | - | 232 | 44 | 1.11E-13 | 5.27 |
| 877807 | 877698 | - | 343 | 98 | 2.33E-04 | 3.50 |
| 878907 | 878821 | - | 153 | 27 | 6.87E-26 | 5.67 |
| 894650 | 894612 | - | 142 | 50 | 0.00608503 | 2.84 |
| 894788 | 894713 | - | 746 | 317 | 0.00241574 | 2.35 |
| 905901 | 905829 | - | 207 | 83 | 0.00361967 | 2.49 |
| 914071 | 914124 | + | 294 | 125 | 0.33385152 | 2.35 |
| 925187 | 925225 | + | 8 | 629 | 1 | 0.01 |
| 929260 | 929195 | - | 852 | 132 | 8.65E-40 | 6.45 |
| 948164 | 948126 | - | 202 | 69 | 0.00198269 | 2.93 |
| 961989 | 961950 | - | 690 | 72 | 1.35E-81 | 9.58 |
| 970732 | 970786 | + | 194 | 76 | 5.80E-04 | 2.55 |
| 975274 | 975240 | - | 235 | 57 | 3.31E-07 | 4.12 |
| 986123 | 986081 | - | 270 | 31 | 4.04E-39 | 8.71 |
| 990155 | 990021 | - | 219 | 58 | 4.61E-09 | 3.78 |
| 997396 | 997357 | - | 287 | 37 | 8.06E-32 | 7.76 |
| 1000320 | 1000251 | - | 29 | 529 | 1 | 0.05 |
| 1000340 | 1000397 | + | 0 | 668 | 1 | 0.00 |
| 1004113 | 1004074 | - | 187 | 150 | 1 | 1.25 |
| 1023050 | 1023019 | - | 0 | 257 | 1 | 0.00 |
| 1035409 | 1035496 | + | 9 | 1332 | 1 | 0.01 |
| 1035887 | 1035927 | + | 5 | 344 | 1 | 0.01 |
| 1044006 | 1043969 | - | 0 | 652 | 1 | 0.00 |
| 1044394 | 1044364 | - | 0 | 281 | 1 | 0.00 |
| 1088434 | 1088474 | + | 172 | 88 | 1 | 1.95 |
| 1091113 | 1091071 | - | 400 | 82 | 1.26E-20 | 4.88 |
| 1095768 | 1095830 | + | 520 | 86 | 1.13E-26 | 6.05 |
| 1100092 | 1100136 | + | 378 | 66 | 7.92E-23 | 5.73 |
| 1100137 | 1100076 | - | 615 | 194 | 0.00327743 | 3.17 |
| 1108221 | 1108183 | - | 177 | 36 | 6.35E-13 | 4.92 |
| 1123469 | 1123365 | - | 0 | 354 | 1 | 0.00 |
| 1133763 | 1133796 | + | 145 | 70 | 0.28435027 | 2.07 |
| 1172604 | 1172667 | + | 18256 | 4069 | 4.22E-09 | 4.49 |
| 1177721 | 1177755 | + | 172 | 112 | 0.79188392 | 1.54 |
| 1184221 | 1184178 | - | 409 | 115 | 1.88E-04 | 3.56 |
| 1188077 | 1187989 | - | 166 | 39 | 4.82E-12 | 4.26 |
| 1195732 | 1195785 | + | 577 | 144 | 1.72E-09 | 4.01 |
| 1215621 | 1215695 | + | 202 | 77 | 0.31017107 | 2.62 |
| 1230989 | 1231029 | + | 344 | 42 | 8.65E-40 | 8.19 |
| 1230994 | 1230933 | - | 166 | 23 | 1.79E-22 | 7.22 |
| 1231146 | 1231196 | + | 180 | 43 | 3.68E-06 | 4.19 |
| 1231178 | 1231064 | - | 1651 | 346 | 3.82E-19 | 4.77 |
| 1231262 | 1231293 | + | 0 | 260 | 1 | 0.00 |
| 1231720 | 1231806 | + | 14 | 1337 | 1 | 0.01 |
| 1231857 | 1231913 | + | 0 | 275 | 1 | 0.00 |
| 1232366 | 1232334 | - | 0 | 225 | 1 | 0.00 |
| 1248943 | 1249028 | + | 1486 | 6193 | 1 | 0.24 |
| 1281294 | 1281332 | + | 154 | 38 | 9.25E-05 | 4.05 |
| 1329031 | 1329066 | + | 128 | 5 | 0 | 25.60 |
| 1372571 | 1372359 | - | 444 | 424 | 0.01297857 | 1.05 |
| 1388634 | 1388595 | - | 154 | 31 | 2.98E-07 | 4.97 |
| 1406383 | 1406346 | - | 1219 | 194 | 3.60E-51 | 6.28 |
| 1428227 | 1428176 | - | 12 | 1091 | 1 | 0.01 |
| 1428660 | 1428717 | + | 150 | 30 | 4.12E-11 | 5.00 |
| 1442292 | 1442051 | - | 302 | 164 | 1 | 1.84 |
| 1442553 | 1442480 | - | 184 | 88 | 1 | 2.09 |
| 1443132 | 1443098 | - | 170 | 53 | 0.01694788 | 3.21 |
| 1443423 | 1443230 | - | 1199 | 284 | 6.54E-14 | 4.22 |
| 1443516 | 1443476 | - | 211 | 74 | 0.03917952 | 2.85 |
| 1446395 | 1446356 | - | 255 | 64 | 2.61E-08 | 3.98 |
| 1458869 | 1458790 | - | 100 | 39 | 0.00178029 | 2.56 |
| 1459040 | 1459078 | + | 782 | 154 | 3.59E-17 | 5.08 |
| 1459075 | 1459034 | - | 268 | 31 | 1.52E-38 | 8.65 |
| 1480609 | 1480648 | + | 182 | 43 | 2.11E-05 | 4.23 |
| 1480679 | 1480472 | - | 347 | 73 | 4.14E-16 | 4.75 |
| 1480960 | 1480999 | + | 213 | 64 | 0.0049693 | 3.33 |
| 1531525 | 1531563 | + | 11 | 640 | 1 | 0.02 |
| 1544827 | 1544942 | + | 1497 | 205 | 1.82E-63 | 7.30 |
| 1547454 | 1547492 | + | 163 | 46 | 8.12E-08 | 3.54 |
| 1551303 | 1551252 | - | 1119 | 226 | 2.33E-16 | 4.95 |
| 1608619 | 1608701 | + | 6665 | 3027 | 1 | 2.20 |
| 1610070 | 1610021 | - | 0 | 498 | 1 | 0.00 |
| 1614227 | 1614259 | + | 0 | 304 | 1 | 0.00 |
| 1617174 | 1617007 | - | 664 | 219 | 7.43E-05 | 3.03 |
| 1621174 | 1621139 | - | 488 | 112 | 1.32E-15 | 4.36 |
| 1629338 | 1629236 | - | 379 | 168 | 0.23543682 | 2.26 |
| 1631059 | 1631018 | - | 27 | 593 | 1 | 0.05 |
| 1642992 | 1642953 | - | 319 | 49 | 4.03E-24 | 6.51 |
| 1644065 | 1644024 | - | 484 | 105 | 4.70E-15 | 4.61 |
| 1644243 | 1644205 | - | 388 | 35 | 1.06E-72 | 11.09 |
| 1660814 | 1660783 | - | 195 | 8 | 3.38E-279 | 24.38 |
| 1732440 | 1732470 | + | 0 | 251 | 1 | 0.00 |
| 1742666 | 1742764 | + | 237 | 66 | 4.82E-07 | 3.59 |
| 1742980 | 1742944 | - | 159 | 36 | 2.18E-09 | 4.42 |
| 1751056 | 1751018 | - | 143 | 74 | 0.54297207 | 1.93 |
| 1752685 | 1752631 | - | 1689 | 280 | 6.94E-30 | 6.03 |
| 1758306 | 1758360 | + | 77 | 321 | 0.16519016 | 0.24 |
| 1759882 | 1759917 | + | 128 | 30 | 1.30E-04 | 4.27 |
| 1762962 | 1762909 | - | 730 | 229 | 0.00110384 | 3.19 |
| 1779946 | 1779990 | + | 1065 | 326 | 6.22E-05 | 3.27 |
| 1790038 | 1790000 | - | 172 | 27 | 3.08E-16 | 6.37 |
| 1791999 | 1791959 | - | 148 | 70 | 0.58616374 | 2.11 |
| 1792287 | 1792228 | - | 267 | 162 | 0.43367241 | 1.65 |
| 1812086 | 1812044 | - | 264 | 366 | 1 | 0.72 |
| 1830723 | 1830870 | + | 0 | 388 | 1 | 0.00 |
| 1848669 | 1848607 | - | 1521 | 249 | 1.30E-31 | 6.11 |
| 1850542 | 1850492 | - | 974 | 414 | 0.08414818 | 2.35 |
| 1850775 | 1850731 | - | 272 | 79 | 1.08E-05 | 3.44 |
| 1855144 | 1855195 | + | 2035 | 298 | 4.52E-42 | 6.83 |
| 1858170 | 1858130 | - | 352 | 123 | 0.06897134 | 2.86 |
| 1881147 | 1881187 | + | 926 | 372 | 0.22218854 | 2.49 |
| 1886063 | 1886013 | - | 271 | 61 | 6.38E-10 | 4.44 |
| 1886209 | 1886157 | - | 252 | 94 | 0.28165051 | 2.68 |
| 1886329 | 1886273 | - | 244 | 35 | 2.43E-26 | 6.97 |
| 1887055 | 1887094 | + | 240 | 29 | 2.08E-30 | 8.28 |
| 1887097 | 1887049 | - | 297 | 47 | 9.59E-22 | 6.32 |
| 1890491 | 1890440 | - | 354 | 73 | 3.93E-17 | 4.85 |
| 1890766 | 1890563 | - | 492 | 95 | 4.92E-22 | 5.18 |
| 1976074 | 1976036 | - | 220 | 225 | 1 | 0.98 |
| 1981300 | 1981261 | - | 339 | 163 | 0.00893816 | 2.08 |
| 1994813 | 1994999 | + | 7571 | 12499 | 0.89159036 | 0.61 |
| 2002043 | 2002081 | + | 497 | 48 | 9.25E-73 | 10.35 |
| 2011273 | 2011380 | + | 264 | 117 | 0.92332932 | 2.26 |
| 2014184 | 2014134 | - | 403 | 139 | 0.02864307 | 2.90 |
| 2015670 | 2015630 | - | 225 | 108 | 1 | 2.08 |
| 2016127 | 2016165 | + | 179 | 70 | 0.22663353 | 2.56 |
| 2036967 | 2036881 | - | 23 | 900 | 1 | 0.03 |
| 2038037 | 2037939 | - | 311 | 156 | 1 | 1.99 |
| 2039123 | 2039088 | - | 163 | 97 | 0.9942772 | 1.68 |
| 2040428 | 2040471 | + | 762 | 133 | 4.72E-24 | 5.73 |
| 2041437 | 2041403 | - | 135 | 103 | 0.04513658 | 1.31 |
| 2042640 | 2042598 | - | 246 | 110 | 1 | 2.24 |
| 2044684 | 2044646 | - | 202 | 66 | 0.03749674 | 3.06 |
| 2045034 | 2044987 | - | 246 | 89 | 0.19768832 | 2.76 |
| 2045700 | 2045658 | - | 232 | 111 | 1 | 2.09 |
| 2046092 | 2046026 | - | 478 | 389 | 0.01114386 | 1.23 |
| 2046215 | 2046171 | - | 247 | 123 | 1 | 2.01 |
| 2057255 | 2057216 | - | 277 | 87 | 3.60E-06 | 3.18 |
| 2061159 | 2061189 | + | 63 | 399 | 0.07965138 | 0.16 |
| 2072238 | 2072270 | + | 170 | 29 | 1.25E-14 | 5.86 |
| 2091321 | 2091366 | + | 259 | 37 | 7.20E-25 | 7.00 |
| 2095489 | 2095528 | + | 183 | 19 | 3.43E-50 | 9.63 |
| 2095588 | 2095534 | - | 1632 | 253 | 2.22E-35 | 6.45 |
| 2101757 | 2101666 | - | 256 | 32 | 4.00E-58 | 8.00 |
| 2102771 | 2102732 | - | 205 | 26 | 4.49E-28 | 7.88 |
| 2111949 | 2112005 | + | 925 | 369 | 0.02161568 | 2.51 |
| 2114338 | 2114301 | - | 168 | 43 | 1.65E-07 | 3.91 |
| 2137369 | 2137408 | + | 302 | 63 | 5.20E-12 | 4.79 |
| 2138303 | 2138394 | + | 5167 | 1074 | 2.00E-18 | 4.81 |
| 2141383 | 2141420 | + | 140 | 7 | 8.91E-167 | 20.00 |
| 2143387 | 2143349 | - | 185 | 25 | 5.79E-28 | 7.40 |
| 2148883 | 2148841 | - | 320 | 231 | 1 | 1.39 |

*: Transcription start and stop sites are shown according to the Neisseria gonorrhoeae FA1090

#: Whether the putative sRNA is expressed from the coding (+) or complement (-) strand

&: The expression level of each putative sRNA in RPKM is shown either during incubation with E6/E7 endocervical cells or in media alone.

**Table S5**

| **Transcription Start** | **Transcription Stop** | **Length** | **Found In** | | | |
| --- | --- | --- | --- | --- | --- | --- |
|  |  |  | **GC INF*** | **GC MED^#^** | **GC + Fe^$^** | **GC – Fe^&^** |
| 36127 | 36075 | 52 | ✓ | ✓ |  |  |
| 52192 | 52153 | 39 | ✓ |  |  |  |
| 52359 | 52327 | 32 | ✓ |  |  |  |
| 68401 | 68441 | 40 | ✓ |  |  |  |
| 69613 | 69550 | 63 |  |  | ✓ |  |
| 69754 | 69718 | 36 |  |  |  | ✓ |
| 74221 | 74259 | 38 | ✓ |  |  |  |
| 75879 | 76037 | 158 | ✓ | ✓ |  |  |
| 76112 | 76048 | 64 | ✓ |  |  |  |
| 81518 | 81473 | 45 | ✓ |  |  |  |
| 100020 | 100105 | 85 | ✓ | ✓ |  |  |
| 102572 | 102533 | 39 | ✓ |  |  |  |
| 103161 | 103231 | 70 | ✓ |  |  |  |
| 108616 | 108576 | 40 | ✓ |  |  |  |
| 111925 | 111959 | 34 | ✓ |  |  |  |
| 130798 | 130832 | 34 | ✓ |  |  |  |
| 137364 | 137325 | 39 | ✓ |  |  |  |
| 164091 | 164042 | 49 | ✓ | ✓ |  |  |
| 207902 | 207863 | 39 | ✓ | ✓ |  |  |
| 259160 | 259200 | 40 | ✓ | ✓ |  |  |
| 260252 | 260298 | 46 | ✓ |  |  |  |
| 260456 | 260513 | 57 | ✓ | ✓ |  |  |
| 263443 | 263410 | 33 | ✓ |  |  |  |
| 270189 | 270150 | 39 | ✓ |  |  |  |
| 300228 | 300287 | 59 | ✓ |  |  |  |
| 329666 | 329704 | 38 | ✓ |  |  |  |
| 341477 | 341518 | 41 | ✓ |  |  |  |
| 423966 | 423925 | 41 | ✓ | ✓ |  |  |
| 424933 | 424973 | 40 | ✓ | ✓ |  |  |
| 425725 | 425693 | 32 | ✓ | ✓ |  |  |
| 441349 | 441302 | 47 | ✓ | ✓ |  |  |
| 453642 | 453761 | 119 | ✓ |  |  |  |
| 471834 | 471942 | 108 |  |  | ✓ | ✓ |
| 496914 | 496876 | 38 | ✓ |  |  |  |
| 511220 | 511259 | 39 | ✓ |  |  |  |
| 557369 | 557424 | 55 | ✓ | ✓ |  |  |
| 587390 | 587351 | 39 | ✓ |  |  |  |
| 587758 | 587717 | 41 | ✓ |  |  |  |
| 588286 | 588020 | 266 | ✓ |  |  |  |
| 599324 | 599360 | 36 | ✓ |  |  |  |
| 600258 | 600311 | 53 | ✓ |  |  |  |
| 621847 | 621886 | 39 | ✓ | ✓ |  |  |
| 637363 | 637413 | 50 | ✓ | ✓ | ✓ | ✓ |
| 639059 | 639120 | 61 | ✓ | ✓ |  |  |
| 640902 | 640863 | 39 | ✓ | ✓ |  |  |
| 711520 | 711571 | 51 | ✓ |  |  |  |
| 745897 | 745954 | 57 | ✓ | ✓ |  |  |
| 785808 | 785909 | 101 | ✓ | ✓ | ✓ |  |
| 823829 | 823788 | 41 | ✓ | ✓ |  |  |
| 827247 | 827284 | 37 | ✓ |  |  |  |
| 846728 | 846793 | 65 | ✓ |  |  |  |
| 862950 | 863310 | 360 | ✓ | ✓ | ✓ | ✓ |
| 870846 | 870753 | 93 | ✓ | ✓ | ✓ | ✓ |
| 876732 | 876688 | 44 | ✓ |  |  |  |
| 876823 | 876785 | 38 | ✓ |  |  |  |
| 877177 | 877123 | 54 | ✓ |  |  |  |
| 877807 | 877698 | 109 | ✓ |  |  |  |
| 878879 | 878840 | 39 | ✓ |  |  |  |
| 894770 | 894731 | 39 | ✓ | ✓ |  | ✓ |
| 914073 | 914124 | 51 | ✓ |  |  |  |
| 929260 | 929195 | 65 | ✓ |  |  |  |
| 948163 | 948130 | 33 | ✓ |  |  |  |
| 961989 | 961950 | 39 | ✓ |  |  |  |
| 970743 | 970785 | 42 | ✓ |  |  |  |
| 975274 | 975243 | 31 | ✓ |  |  |  |
| 986123 | 986084 | 39 | ✓ |  |  |  |
| 990155 | 990063 | 92 | ✓ |  |  |  |
| 997396 | 997357 | 39 | ✓ |  |  |  |
| 1035409 | 1035469 | 60 |  |  | ✓ | ✓ |
| 1088433 | 1088477 | 44 | ✓ |  |  |  |
| 1091113 | 1091071 | 42 | ✓ |  |  |  |
| 1095768 | 1095817 | 49 | ✓ |  |  |  |
| 1100131 | 1100092 | 39 | ✓ | ✓ |  |  |
| 1116047 | 1116079 | 32 | ✓ |  |  |  |
| 1172604 | 1172666 | 62 | ✓ | ✓ |  |  |
| 1177721 | 1177754 | 33 | ✓ |  |  |  |
| 1195742 | 1195785 | 43 | ✓ | ✓ |  |  |
| 1215651 | 1215694 | 43 | ✓ |  |  |  |
| 1230972 | 1230934 | 38 | ✓ |  |  |  |
| 1230989 | 1231029 | 40 | ✓ |  |  |  |
| 1231157 | 1231187 | 30 | ✓ |  |  |  |
| 1231171 | 1231109 | 62 | ✓ | ✓ |  |  |
| 1231686 | 1231950 | 264 |  |  | ✓ |  |
| 1231720 | 1231780 | 60 |  |  |  | ✓ |
| 1248743 | 1248826 | 83 | ✓ | ✓ | ✓ |  |
| 1248943 | 1249016 | 73 | ✓ | ✓ | ✓ | ✓ |
| 1249132 | 1249180 | 48 | ✓ |  |  |  |
| 1300487 | 1300450 | 37 | ✓ |  |  |  |
| 1305820 | 1305768 | 52 | ✓ |  |  |  |
| 1372551 | 1372362 | 189 | ✓ | ✓ |  |  |
| 1386502 | 1386435 | 67 | ✓ |  |  |  |
| 1426808 | 1426846 | 38 | ✓ |  |  |  |
| 1428226 | 1428167 | 59 |  |  | ✓ |  |
| 1442289 | 1442052 | 237 | ✓ |  |  |  |
| 1442546 | 1442502 | 44 | ✓ |  |  |  |
| 1443403 | 1443238 | 165 | ✓ | ✓ | ✓ |  |
| 1443516 | 1443477 | 39 | ✓ |  |  |  |
| 1446395 | 1446356 | 39 | ✓ |  |  |  |
| 1459072 | 1459036 | 36 | ✓ |  |  |  |
| 1480683 | 1480472 | 211 | ✓ |  |  |  |
| 1480960 | 1480998 | 38 | ✓ |  |  |  |
| 1486365 | 1486403 | 38 | ✓ |  |  |  |
| 1531309 | 1531359 | 50 |  |  | ✓ |  |
| 1544828 | 1544942 | 114 | ✓ | ✓ |  |  |
| 1547454 | 1547492 | 38 | ✓ |  |  |  |
| 1551292 | 1551253 | 39 | ✓ | ✓ |  |  |
| 1557957 | 1557918 | 39 | ✓ |  |  |  |
| 1608639 | 1608713 | 74 | ✓ |  |  |  |
| 1617152 | 1617072 | 80 | ✓ | ✓ |  |  |
| 1621174 | 1621139 | 35 | ✓ |  |  |  |
| 1631047 | 1630998 | 49 |  |  |  | ✓ |
| 1642992 | 1642953 | 39 | ✓ |  |  |  |
| 1643726 | 1643646 | 80 |  |  | ✓ |  |
| 1644065 | 1644026 | 39 | ✓ |  |  |  |
| 1644243 | 1644205 | 38 | ✓ |  |  |  |
| 1716392 | 1716446 | 54 | ✓ | ✓ |  |  |
| 1742666 | 1742709 | 43 | ✓ | ✓ |  |  |
| 1752678 | 1752637 | 41 | ✓ | ✓ |  |  |
| 1759881 | 1759920 | 39 | ✓ |  |  |  |
| 1762955 | 1762916 | 39 | ✓ | ✓ |  |  |
| 1779946 | 1779990 | 44 | ✓ | ✓ |  |  |
| 1796157 | 1796060 | 97 |  |  |  | ✓ |
| 1830718 | 1830788 | 70 |  |  |  | ✓ |
| 1848653 | 1848613 | 40 | ✓ | ✓ |  |  |
| 1850541 | 1850494 | 47 | ✓ | ✓ |  |  |
| 1850772 | 1850731 | 41 | ✓ |  |  |  |
| 1855144 | 1855195 | 51 | ✓ | ✓ |  |  |
| 1858170 | 1858130 | 40 | ✓ |  |  |  |
| 1872877 | 1872914 | 37 |  |  |  | ✓ |
| 1881147 | 1881187 | 40 | ✓ |  |  |  |
| 1886060 | 1886012 | 48 | ✓ |  |  |  |
| 1886212 | 1886159 | 53 | ✓ |  |  |  |
| 1887055 | 1887093 | 38 | ✓ |  |  |  |
| 1887096 | 1887055 | 41 | ✓ |  |  |  |
| 1890491 | 1890441 | 50 | ✓ |  |  |  |
| 1890664 | 1890703 | 39 | ✓ | ✓ |  |  |
| 1890668 | 1890563 | 105 | ✓ | ✓ |  |  |
| 1910182 | 1910246 | 64 |  |  | ✓ |  |
| 1924431 | 1924792 | 361 | ✓ | ✓ | ✓ | ✓ |
| 1976085 | 1976037 | 48 | ✓ |  |  | ✓ |
| 1981300 | 1981261 | 39 | ✓ |  | ✓ |  |
| 1989247 | 1989163 | 84 | ✓ | ✓ |  |  |
| 2002043 | 2002081 | 38 | ✓ |  |  |  |
| 2011273 | 2011313 | 40 | ✓ |  |  |  |
| 2016129 | 2016159 | 30 | ✓ |  |  |  |
| 2036967 | 2036907 | 60 |  |  | ✓ |  |
| 2038034 | 2037941 | 93 | ✓ | ✓ |  |  |
| 2040428 | 2040471 | 43 | ✓ | ✓ |  |  |
| 2041432 | 2041400 | 32 | ✓ |  |  |  |
| 2042637 | 2042600 | 37 | ✓ |  |  |  |
| 2044189 | 2044154 | 35 |  | ✓ |  |  |
| 2044682 | 2044646 | 36 | ✓ |  |  |  |
| 2045035 | 2044987 | 48 | ✓ |  |  |  |
| 2046080 | 2046027 | 53 | ✓ |  |  |  |
| 2046214 | 2046172 | 42 | ✓ |  |  |  |
| 2057255 | 2057216 | 39 | ✓ |  |  |  |
| 2091326 | 2091366 | 40 | ✓ |  |  |  |
| 2095581 | 2095540 | 41 | ✓ | ✓ |  |  |
| 2101757 | 2101669 | 88 | ✓ |  |  |  |
| 2102771 | 2102732 | 39 | ✓ |  |  |  |
| 2111953 | 2112001 | 48 | ✓ |  |  |  |
| 2137369 | 2137408 | 39 | ✓ |  |  |  |
| 2138303 | 2138392 | 89 | ✓ |  |  |  |
| 2143387 | 2143349 | 38 | ✓ |  |  |  |

*: Check indicates sRNA expressed during incubation of *N. gonorrhoeae* with endocervical cells

#: Check indicates sRNA expressed during incubation of *N. gonorrhoeae* in KSFM media alone

$: Check indicates sRNA expressed during incubation of *N. gonorrhoeae* in CDM with 100 μM ferric nitrate

&: Check indicates sRNA expressed during incubation of *N. gonorrhoeae* in CDM with 100 μM desferal
